# Potassium Retention under Salt Stress Is Associated with Natural Variation in Salinity Tolerance among *Arabidopsis* Accessions

**DOI:** 10.1371/journal.pone.0124032

**Published:** 2015-05-19

**Authors:** Yanling Sun, Xiangpei Kong, Cuiling Li, Yongxiu Liu, Zhaojun Ding

**Affiliations:** 1 The Key Laboratory of Plant Cell Engineering and Germplasm Innovation, College of Life Sciences, Shandong University, 27 Shanda South Road, Jinan, 250100, Shandong, China; 2 The Key Laboratory of Plant Molecular Physiology, Institute of Botany, Chinese Academy of Sciences, 100093, Beijing, China; Indiana University, UNITED STATES

## Abstract

Plants are exposed to various environmental stresses during their life cycle such as salt, drought and cold. Natural variation mediated plant growth adaptation has been employed as an effective approach in response to the diverse environmental cues such as salt stress. However, the molecular mechanism underlying this process is not well understood. In the present study, a collection of 82 *Arabidopsis thaliana* accessions (ecotypes) was screened with a view to identify variation for salinity tolerance. Seven accessions showed a higher level of tolerance than Col-0. The young seedlings of the tolerant accessions demonstrated a higher K^+^ content and a lower Na^+^/K^+^ ratio when exposed to salinity stress, but its Na^+^ content was the same as that of Col-0. The K^+^ transporter genes *AtHAK5*, *AtCHX17* and *AtKUP1* were up-regulated significantly in almost all the tolerant accessions, even in the absence of salinity stress. There was little genetic variation or positive transcriptional variation between the selections and Col-0 with respect to Na^+^-related transporter genes, as *AtSOS* genes, *AtNHX1* and *AtHKT1;1*. In addition, under salinity stress, these selections accumulated higher compatible solutes and lower reactive oxygen species than did Col-0. Taken together, our results showed that natural variation in salinity tolerance of *Arabidopsis* seems to have been achieved by the strong capacity of K^+^ retention.

## Introduction

Salinity is one of the most serious environmental constraints to plant growth and crop productivity [1]. It is responsible for a combination of ionic and osmotic stresses, which together inhibit leaf expansion, restrict photosynthesis and limit the accumulation of biomass [2–3]. The adaptive response by many plants to salinity stress includes the active exclusion of sodium (Na^+^) ions and/or their sequestration into the vacuole, the production of compatible solutes and reactive oxygen species (ROS) detoxification [1, 4–8].

An increasing number of genes specifying relevant salt tolerance have been identified in recent years in both the model plant *Arabidopsis thaliana* (hereafter *Arabidopsis*) and other species. One of the most prominent of these belongs to the Salinity Overly Sensitive (SOS) signaling pathway, which has been proposed as a key component in the process of Na^+^ exclusion and the maintenance of ion homeostasis [9–10]. The SOS pathway genes comprise *SOS1* (encoding a plasma membrane Na^+^/H^+^ antiporter, also known as *NHX7*), *SOS2* (a Ser/Thr kinase) and *SOS3* (a Ca^2+^-binding protein), which transduce a salt stress-induced Ca^2+^ signal to regulate to Na^+^ efflux at cellular level and control long distance transport of Na^+^ from root to shoot [6, 11–20]. The over-expression of *SOS1* in *Arabidopsis* has been shown to confer a heightened level of salinity tolerance [21–23]. The high-affinity K^+^ transporters (HKT1;1) is a further determinant of salinity tolerance in *Arabidopsis*, which controls Na^+^ entry into plant roots and Na^+^ recirculation by the phloem [24–30]. Besides, the tonoplast-localized Na^+^/H^+^ exchanger (NHX1) catalyzes Na^+^ sequestration in vacuoles [6, 31], and its C terminus is correlated with the regulation of the antiporter selectivity [32]. The importance of Na^+^ compartmentation to the vacuole by overexpressing *AtNHX1* has been shown by improved performance in salt-stressed transgenic tomato [33], brassica [34], rice [35].

Potassium (K^+^) is also a major factor in resistance to salinity, drought and fungal diseases in plant [36–38]. Previously the growing bulk of evidence has demonstrated that there is a strong positive correlation between the ability to retain K^+^ and overall plant salt tolerance when challenged with NaCl in several species including barley [39–40] and wheat [41]. So far, a large number of genes encoding K^+^ transporters and channels have been identified for high/low-affinity K^+^ uptake in *Arabidopsis* [42–46]. The high affinity K^+^ transporter 5 (*HAK5*), belongs to KUP/HAK/KT families, located in the epidermis of primary and lateral roots, has been identified as high-affinity transporters [47–49], and is associated with K^+ ^accumulation by genetic mapping of natural variations in K^+^ concentration in *Arabidopsis* [50]. In addition, the K^+^ uptake transporter 1 (*KUP1*) is referred to as a dual- or high-affinity transporter depending on the expression system [51–52]. Moreover, the cation/H^+^ exchanger 17 (*CHX17*), one of 28 *CHX* gene families, is also known to be induced in response to salinity, and may contribute towards K^+^ homeostasis [53].

*Arabidopsis* accessions are widely distributed and known to vary considerably with respect to their adaption to various surrounding environments. In response, considerable genetic variations and adaptive traits of them have also been described for their level of resistance/tolerance to both biotic and abiotic stress [54–58], as well as the various developmental traits such as the flowering time [59], circadian rhythms [60], trichome cell fate determination [61], seed size and dormancy [62]. Along with the allelic variation at the gene sequence exposed by ongoing re-sequencing of the *Arabidopsis* genome [63], global transcriptomic analyses have revealed that the response to several abiotic and biotic stresses can vary between *Arabidopsis* accessions at the level of specific transcript abundance [55, 64–65]. Some of the genes responsible for this variation have been successfully identified by genetic means [54, 66–69]. An extensive characterization of the existing natural genetic variation for salinity tolerance in *Arabidopsis* has identified a few quantitative trait loci (QTLs) and genomic regions as harboring relevant genes [56, 58]. Furthermore, DNA microarray based genotyping was utilized to identify *AtHKT1;1* associated with salt tolerance as the casual locus driving the natural variation in shoot Na^+^ accumulation in Ts-1 and Tsu-1 accessions and it also been found that a coastal cline in sodium accumulation in *Arabidopsis* is driven by natural variation of *AtHKT1;1* [68, 70]. However, salt tolerance is a complex trait and controlled by multiple genes, and involved with various molecular and biochemical mechanisms. So far, how *Arabidopsis* accessions achieve a particular level of salt tolerance in nature is not well known.

Here, we describe the variation for salinity tolerance shown by a set of 82 *Arabidopsis *accessions, and the identification of seven particularly tolerant accessions by comparison with a reference accession Col-0 as reported previously [71]. An analysis of the behavior of these salt-tolerant accessions has demonstrated that the high K^+ ^content is a key factor for their adaptive response, and some relevant K^+^ transporter genes may play active roles for maintaining high K^+^ contents in these tolerant accessions, and our results provide important information for understanding the mechanisms underlying natural variation mediated salt tolerance in *Arabidopsis*. In addition, these selections also accumulated a higher compatible solutes concentration and a lower ROS than did Col-0 under salinity stress.

## Materials and Methods

### Plant materials and growing conditions

The set of 82 *Arabidopsis* accessions (S1 Table) screened for salinity tolerance comprised: Amel-3 (V10530), Asc-1 (CS22163), Be-0 (CS6613), Benk-3 (W10286), Blh-1 (CS6645), Borde (W10290), Boxmeer (W10545), Bs-1 (CS6627), Cal-0 (CS6659), Cam-3 (W40296), Can-0 (CS1065), Churchill (CS1072), Cnt (CS1635), Col-0 (A22625), Csh-1 (CS22419), Deil-1 (W10293), Driel-1 (CS75949), Ei-2 (CS6689), Esp-1 II (CS40021), Everlaan voor (W10170), Fet-1 (W40284), Fet-6 (W40286), Fi-1 (CS6705), Got-1 (CS22277), Hs-1 (CS22351), Ka-0 (CS6752), Kil-0 (CS6754), Kno-1 (CS22401), Kyo-1 (V10372), Kz-2 (CS22436), Lac-2 (W40288), Lac-4 (W40289), Lim (CS8070), Lm-2 (CS6784), Looe-2 (V10540), Lth-1 (CS22363), Mar 2–4 (W40278), Marb-1 (CS75968), Mh-1 (CS6793), Mib-3 (V40305), Mir-0 (N1379), Mog-1 (V40292), Mog-11 (V40293), Mog-12 (V40294), Nd-1 (CS1636), No-0 (CS1394), Oerd-2 (W10299), Oerd-4 (W10040), O-17 (V10451), Ovliel-1 (V10440), Oy-0 (N1437), Pa-1 (N1439), Penz-2 (V10543), Polp-1 (V10536), Pyl (V10370), Ren-1 (CS22253), Rhen-3 (V10498), Rhen-4 (V10499), Rsch-0 (CS6848), Sah-0 (CS6917), Sav-0 (N1514), Seat-1 (CS22341), Sf-2 (CS1516), Sij-2 (CS9655), Sij-4 (CS9656), St.yues-lik (V10537), Ta-0 (N1549), Tanz-1 (CS75924), Te-0 (CS6918), Terlet-1 (CS75950), Treb-1 (V10542), Ts-1 (A22647), Tsu-1 (N1640), Vliel-1 (V10438), Wag-11 (W10295), Wag-13 (V10428), Wei-1 (CS6182), Wil-1 (N1595), Ws-0 (N1595), Ws-2 (CS2360), Wt-4 (CS6895) and Wt-5 (CS6896). The seeds were surface-sterilized overnight in chlorine which was induced by NaClO and HCl, then dried for 20 min before plating on solidified Murashige and Skoog (MS) medium (0.8% (w/v) agar, pH 5.7) supplemented with 1% (w/v) sucrose. The seeds were exposed to 4°C in the dark for 48 h, and then removed to a 16 h photoperiod (350 μmol m^-2^ s^-2^) at 23°C and 60% relative humidity.

### Salinity tolerance assays

Seedlings were exposed to salinity stress in three different ways. (1) Three-day-old seedlings germinated on solidified MS medium (see above) were transferred to a similar medium supplemented with 0, 150, 180 or 200 mM NaCl, and allowed to grow for a further six days under the conditions described above. (2) Five-day-old seedlings were floated on a nylon mesh suspended over liquid MS medium for three days, and exchanged thereafter on a daily basis over the next three days by liquid MS medium containing 0 or 200 mM NaCl. (3) Nine-day-old seedlings germinated on solidified MS medium were transplanted into an autoclaved mixture of perlite/vermiculite/peat (1/1/3, v/v) and left to establish for three weeks, after which they were watered to capacity every two days for the next twenty days with 500 mM NaCl (as a control, a set of similar plants was given just water).

### Growth parameters, chlorophyll and malondialdehyde (MDA) content

Seeds was germinated and grown on solidified MS medium as above. To assay root growth, three-day-old seedlings were transferred to an MS medium containing 0 or 150 mM NaCl and left to grow for a further nine days. Root length was measured from digital images using ImageJ software (http://imagej.nih.gov/ij/). Measurements were expressed in the form of the ratio between the mean growth of primary root lengths achieved in the salinity treatment and the control treatment, based on at least 20 seedlings. Salinity-induced chlorosis of the cotyledons was assessed by eye after exposing three-day-old seedlings to a medium containing 200 mM NaCl for six days. Leaf chlorophyll content was measured on seedlings which had been germinated for three days on solidified MS medium, then transferred to the same medium containing 200 mM NaCl for a further five days. The leaf material was homogenized in cold 96% ethanol, and its chlorophyll content determined following [72]. The MDA assay was carried out on six-day-old seedlings grown on MS medium, then transferred to a liquid MS medium containing 0 or 200 mM NaCl for 36 h. MDA content was measured using a modified thiobarbituric acid (TBA) method [29]. Each experiment was replicated three times.

### Measurements of Na^+^ and K^+^ content

Three-day-old seedlings grown on solidified MS medium were transferred to a liquid MS containing 0 or 200 mM NaCl. Plants were harvested after 12 h of salt treatment. The whole plants of control and salt-treated group for each accession were rinse separately in sterile distilled water timely, then dried quickly on the filter paper and fresh weights were recorded. Samples were digested in 1% (w/v) nitric acid and 30% (w/v) hydrogen peroxide, of which the ratio is 1:1 (v/v). Na^+^ and K^+^ contents were measured using flame atomic absorption spectrophotometer (AA-7000, Japan) following [73]. The results presented are the average and standard error of the mean (SE) for three biological replicates.

### Ion flux measurement

Net K^+^ fluxes were measured at the YoungerUSA (Xuyue Beijing) NMT Service Center by using Non-invasive Micro-test Technology (NMT100 Series^①^, YoungerUSA LLC, Amherst, MA01002, USA; Xuyue (Beijing) Sci. & Tech. Co., Ltd., Beijing, China) and iFluxes/imFluxes 1.0 (YoungerUSA, LLC, Amherst, MA 01002, USA) Software.

Pre-pulled and silanized glass micropipettes (Φ5±1μm, XY-DJ-01, YoungerUSA) were first filled with a backfilling solution (100 mM KCl) to a length of approximately 1.0 cm from the tip. The micropipettes were front filled with 180 μm columns of selective liquid ion-exchange cocktails (K^+^ LIX: XY-SJ-K, Sigma 60031, YoungerUSA). An Ag/AgCl wire electrode holder (XY-DJGD, YoungerUSA) was inserted in the back of the electrode to make electrical contact with the electrolyte solution. YG003-Y05 (YoungerUSA) was used as the reference electrode. Prior to the flux measurement, the microelectrodes were calibrated with cultural media with different concentrations of K^+^, 0.05 mM and 0.5 mM respectively. Only electrodes with a Nernstian slope > 53 mV/decade were used in this study. The concentration gradients of specific K^+^ ions were found by means of selective micro-electrodes “vibrating” repeatedly between two points in a user predefined fashion. The molecular/ionic fluxes are calculated based on Fick’s law of diffusion: *J* = −*D*_*0*_·(*dc/dx*), where *J* is the ion flux (unit: pico mole cm^-2^ s^-1^), *dc* is its concentration gradient, *dx* is the distance the microelectrode moved repeatedly from one point to another perpendicular to the surfaces of samples at a frequency of ca. 0.3 Hz and usually between 5–35 μm, and *D*_*0*_ is its diffusion constant. The direction of the flux is derived from Fick’s law of diffusion.

To measure ion fluxes, 4-day-old *Arabidopsis* accession seedlings were pretreated with 100 mM NaCl for 0 or 3 h in liquid MS medium (without sucrose, pH 5.7). The seedlings were then washed 3 times with redistilled water and the roots were immobilized on the bottom of the chamber containing the 5–10 mL measuring buffer (0.1 mM KCl, 0.1 mM MgCl_2_, 0.1 mM NaCl, 0.1 mM CaCl_2_, 0.3 mM MES, pH 5.7) for 20 minutes. The control group was subjected to the same conditions for 10 minutes. The concentration gradients of the K^+^ ion were measured at the mature root zone (> 2 mm from the root tip, where the first root hair appeared) by moving the ion-selective microelectrode between two positions close to the plant root with a distance of 20 μm. 6 minutes of continuous recording were performed at each measurement point. The steady-state K^+^ flux rates were expressed as the mean of the measured points of 6–15 seedlings and the error bars indicated the SD.

### Measurements of proline, soluble sugar and soluble protein content

The fresh weight of six-day-old seedlings raised on solidified MS medium, then exposed to a liquid MS medium containing 0 or 200 mM NaCl for 36 h, was measured and the material was snap-frozen in liquid nitrogen. Proline was extracted and estimated using the ninhydrin acid reagent method, employing proline as the standard [74]. The soluble sugar and soluble protein content of three-day-old seedlings raised on solidified MS medium and then exposed to a liquid MS medium containing 0 or 200 mM NaCl for 12 h was measured by first obtaining their fresh weight, then following the protocols described by Bailey and Bradford respectively [75–76]. At least 15 plants were included in each of the three replicate measurements.

### Determination of ROS content and superoxide dismutase (SOD) activity

Five-day-old seedlings raised in solidified medium were transferred to MS medium containing 150 mM NaCl for ten days. The superoxide content of the extracted tissue was assessed using nitroblue tetrazolium (NBT) staining [77] and that of H_2_O_2_ by 3, 3’-diaminobenzidine (DAB) staining [78]. The necessary reagents were purchased from Sigma-Aldrich (SAINT LOUIS, USA).

For the analysis of SOD isozymes, three-day-old seedlings raised on solidified MS medium were transferred to a liquid MS medium containing 100 mM NaCl for 0, 3 and 9 h. The seedling was snap-frozen and ground to a powder, from which soluble protein was extracted by homogenization in the mix of 50 mM potassium phosphate buffer (pH 7.8), 1 mM EDTA, 1% w/v polyvinylpyrrolidone. The homogenate was centrifuged (15 000 g × 10 min) at 4°C and the supernatant assayed for SOD activity in accordance with [79]. The reactions were quantified spectrophotometrically at 560 nm.

### Re-sequencing of *AtSOS1-3*, *AtNHX1* and *AtHKT1;1*

Genomic DNA was extracted from the seven selected accessions and Col-0 plants using a CTAB-based protocol [57] and PCR-amplified using the primer sequences given in S2 Table. The resulting amplifications were purified and sequenced.

### Total RNA extraction and Quantitative real-time PCR (qRT-PCR) analysis

Five-day-old seedlings were exposed to a liquid MS medium containing 100 mM NaCl for 0, 3 or 6 h. The tissue was snap-frozen, ground to a powder and used as a source of RNA by extracting with an RNeasy Plant Mini kit (Qiagen, Germany). The resulting total RNA was treated with RNase-free DNase I (Fermentas, USA) and then reverses transcribed using Superscript III reverse transcriptase (Invitrogen, Carlsbad, CA, USA). qRT-PCRs were performed in Bio-RAD CFX Connect Real-time PCR Detection System (Bio-Rad, Hercules, CA) based on the SYBR Green I Master kit (Roche, Grossbasel, Switzerland) according to the manufacturer’s instructions. The *Arabidopsis ACTIN2* gene was used as the reference sequence, and transcript abundances were determined by the 2^-△△CT^ method [80]. The PCR temperature regime comprised a 95°C/3 min denaturation, followed by 40 cycles of 95°C/10 s, 59°C/30 s, 72°C/10 s. The quality of the PCR reactions was estimated based on melting curves. Data were analyzed using CFX Manager software. All primer sequences used are given in S3 Table. Each qRT-PCR was based on three biological replicates and three technical replicates.

### Statistical analyses

The data were reported as mean values ± standard error (SE) from at least three independent experiments with three replicates. All data were analyzed using Student’s *t*-test. Significance between one of the selected accessions and Col-0 accession was determined by *P*-values (* means *P* < 0.05, ** means *P* < 0.01).

Sequence data from this article can be found in the GenBank/EMBL data libraries under accession numbers *AtSOS1* (AT2G01980), *AtSOS2* (AT5G35410), *AtSOS3* (AT5G24270), *AtNHX1* (AT5G27150), *AtHKT1;1* (AT4G10310), *AtHAK5* (AT4G13420), *AtCHX17* (AT4G23700), *AtKUP1* (AT2G30070), *AtGORK* (AT5G37500), *AtProDH1* (AT3G30775), *AtP5CDH* (AT5G62530), *AtP5CR* (AT5G14800), *AtP5CS1* (AT2G39800), *AtP5CS2* (AT3G55610), *AtCSD1* (AT1G08830), *AtCAT2* (AT4G35090), *AtAPX2* (AT3G09640), *AtZAT10* (AT1G27730), *AtZAT12* (AT5G59820) and *AtACTIN2* (AT3G18780).

## Results

### Identification of salinity tolerant accessions and the physiological basis of their tolerance

**Fig 1:**
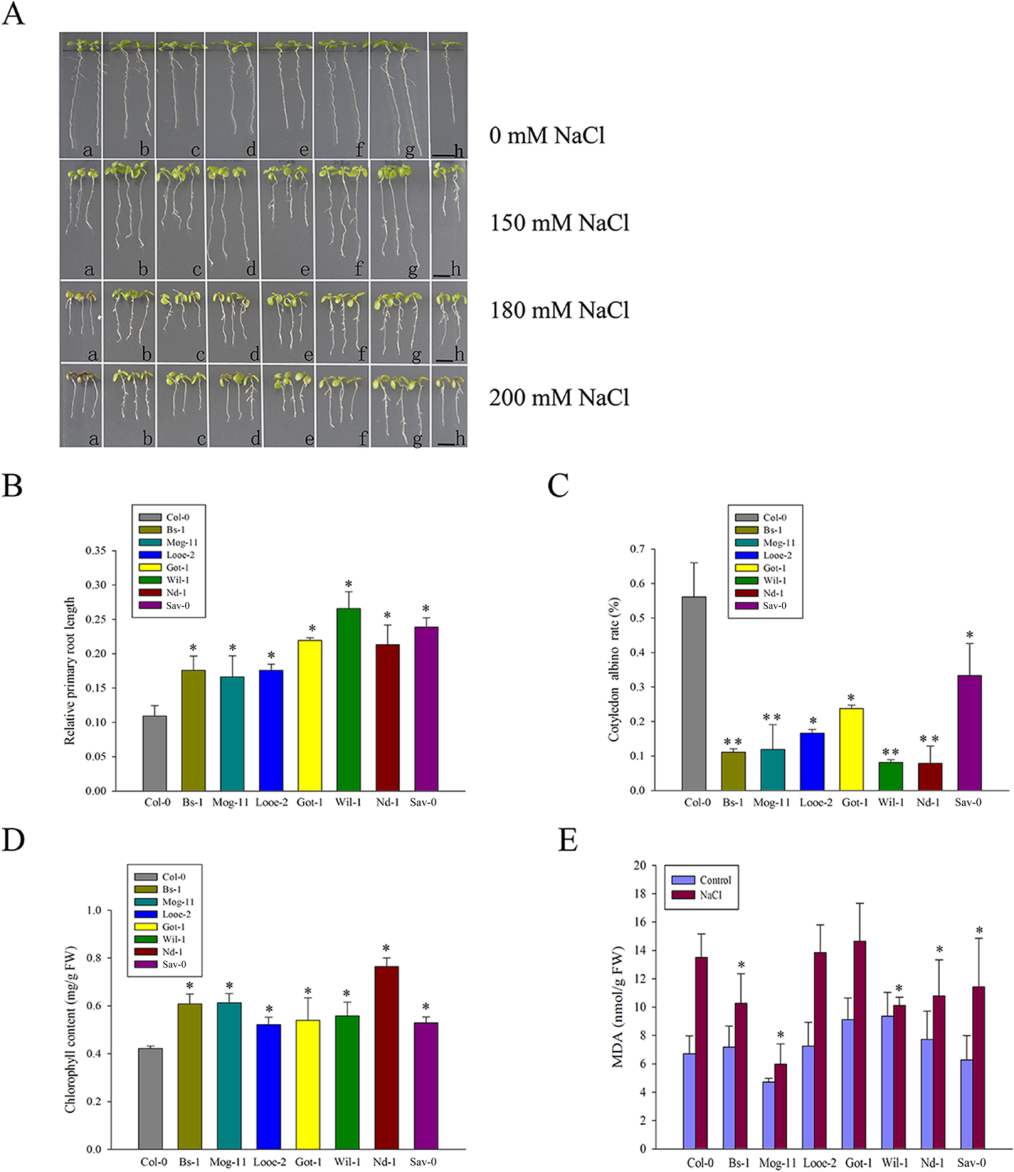
The effect of salinity stress on the selected accessions. (A) Col-0 and the selected accessions seedlings exposed for 6 days to a medium containing 0 mM (uppermost panel), 150 mM (upper panel), 180 mM (middle panel) and 200 mM (lower panel) NaCl. a: Col-0; b: Mog-11; c: Looe-2; d: Wil-1; e: Bs-1; f: Nd-1; g: Sav-0; h: Got-1. Bar = 0.5 cm. (B) Elongation of the primary root of Col-0 and the selected accessions after a 9 day exposure to 0 or 150 mM NaCl. Values represent the mean percentages of root length achieved in the absence of NaCl stress. (C) Bleaching of the cotyledons of Col-0 and the selected accessions exposed for 6 days to 200 mM NaCl. (D) Chlorophyll content of Col-0 and the selected accessions exposed to 200 mM NaCl for 5 days. (E) MDA content of Col-0 and the selected accessions exposed to 0 or 200 mM NaCl for 36 h. All experiments were run in triplicate and involved at least 20 seedlings per assay. Values given as mean ± SE (*n =* 3). Significant differences from the performance of Col-0 accession are indicated by * or ** (*P* < 0.05, *P* < 0.01).

Eighty-two *Arabidopsis* accessions from natural populations, collected in islands or coastal habitats from Europe, Asia, East Africa and North America, were challenged by growing the plants on a medium containing 150 or 200 mM NaCl (S1 Table). With Col-0 as the reference accession, nineteen accessions showed severe sensitivity and the primary root of them were inhibited extremely under 150 mM NaCl (S1 Table). The other accessions showed less growth inhibition than Col-0 in response to 150 mM NaCl, however, under 200 mM NaCl, some were not able to withstand, showed increased chlorosis and even died, in view of this, these accessions was not considered as the salt-tolerant ones (S1 Table). Finally, seven entries from Europe comprised of Bs-1 (Switzerland), Mog-11 (France), Looe-2 (UK), Got-1 (Germany), Wil-1 (Russia), Nd-1 (Germany), and Sav-0 (Czech Rep), were selected as the salt-tolerant ones in this study. To further evaluate their response to salt stress, agar medium supplemented with four different NaCl concentrations (0, 150, 180 and 200 mM) were used, as a result, the selected accessions exhibited obvious tolerant phenotypes (Fig 1A). Moreover, the presence of 150 mM NaCl reduced both the rate of elongation of the primary root and the chlorophyll content, but both traits were less affected in the seven selected accessions than in Col-0 (Fig 1B–1D). In the presence of 200 mM NaCl, the selections had lower cotyledon albino rate compared to Col-0 (Fig 1C). Furthermore, the MDA were accumulated in all tested accessions when subjected to salt stress, but the levels of MDA in most of the selections were still less than in Col-0 (Fig 1E). In addition, the improved tolerance of the selections was also confirmed by using soil or hydroponics assay, as shown in S1 Fig, Col-0 was observed an increased chlorosis compared with the tolerant ones.

### *Arabidopsis* salinity tolerance depended more on K^+^ contents

**Fig 2:**
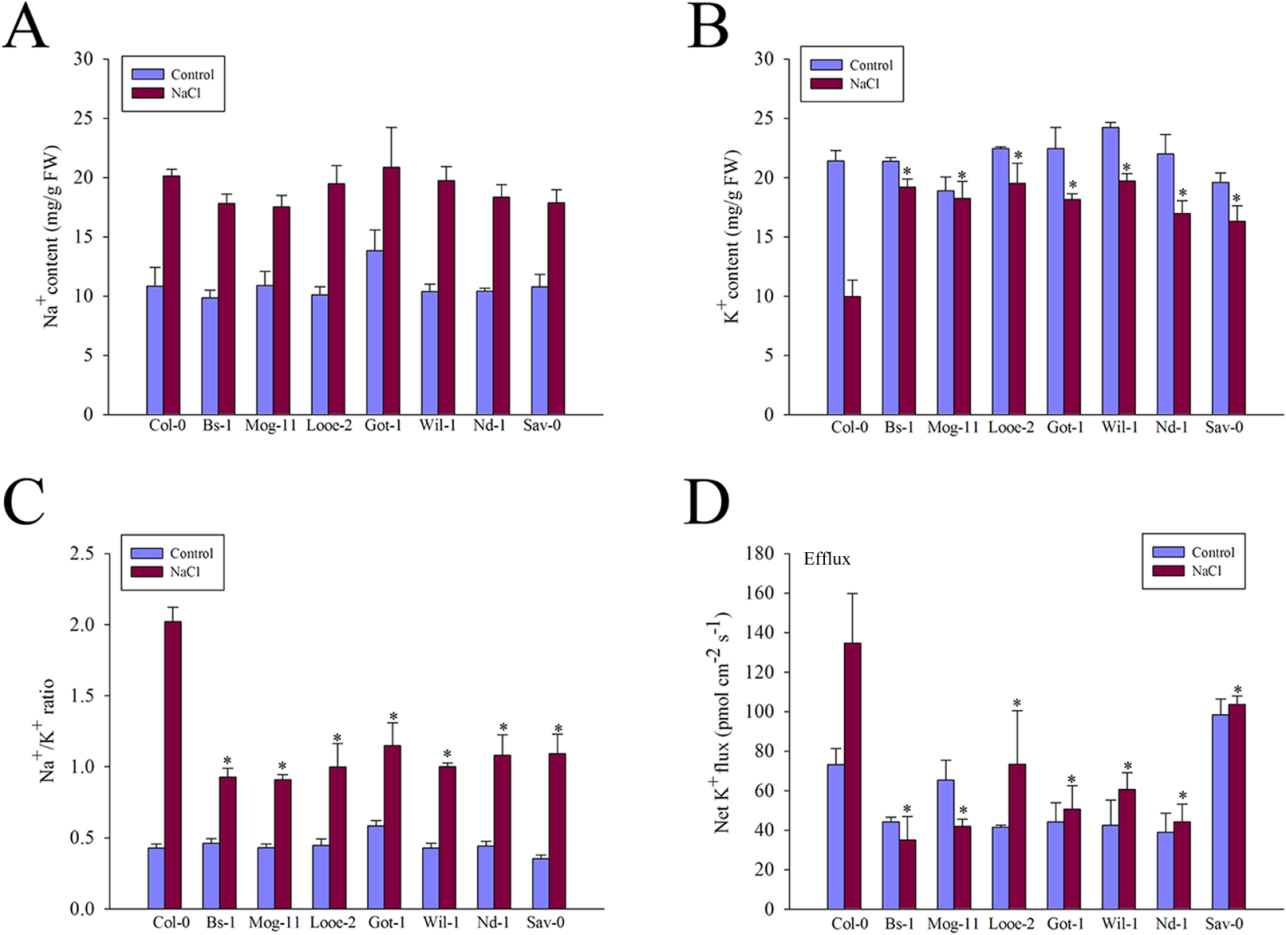
Changes in ion contents, Na^+^/K^+^ ratio and net K^+^ fluxes of Col-0 and the selected accessions. The whole plants treated with 0 or 200 mM for 12 h were collected and subjected to the determination. (A) Na^**+**^ content, (B) K^**+**^ content, (C) Na^**+**^/K^**+**^ ratio and (D) Net K^**+**^ fluxes in root tips. Different net K^**+**^ fluxes were measured respectively in mature zones of the accessions exposed to 100 mM NaCl for 0 or 3 h, Values are mean ± SE (*n* = 6–15 seedlings) from three independent experiments. Significant differences from the performance of Col-0 accession are indicated by * (*P* < 0.05).

In order to investigate whether the change of Na^+^ and K^+^ contents is related to the underlying salinity tolerance in the selected *Arabidopsis* accessions, the amount of total plant Na^+^ and K^+^ contents were measured. The results showed that there was no discernible difference in the Na^+^ contents of the whole plants harvested from the selected accessions and Col-0, whether or not the plants had been exposed to salinity (Fig 2A). Despite the high level of Na^+^ in the selected accessions, they were able to tolerate the stress, which indicated that Na^+^ exclusion was not a special factor of their elevated salinity tolerance. Although the K^+^ contents of all seven accessions were reduced by salinity stress, they were still able to maintain a higher level than that was achieved by Col-0 (Fig 2B), resulting in a lower Na^+^/K^+^ ratio (Fig 2C).

### K^+^ fluxes response

Significant differences were found when net K^+^ fluxes were measured from the mature root zone in response to 0 or 100 mM NaCl treatment (Fig 2D). Although all accessions showed significant net K^+^ efflux in response to salinity, the magnitude of this efflux was significantly different between the tolerant ones and Col-0. As shown in Fig 2D, average K^+^ efflux after salt application was only 30–70 pmol m^-2^ s^-1^ for the salt-tolerant accessions (Bs-1, Mog-11, Looe-2, Got-1, Wil-1 and Nd-1) and about 100 pmol m^-2^ s^-1^ for Sav-0, however, Col-0 was up to 130–140 pmol m^-2^ s^-1^ K^+^ efflux rate after salt stress. These results showed that these tolerant selections had lower K^+^ effluxes than the sensitive Col-0, which result in the strong K^+^ retention and enhanced K^+^ contents in these selections after salinity stress.

### High transcript levels of K^+^ transporter genes in salt-tolerant accessions

**Fig 3:**
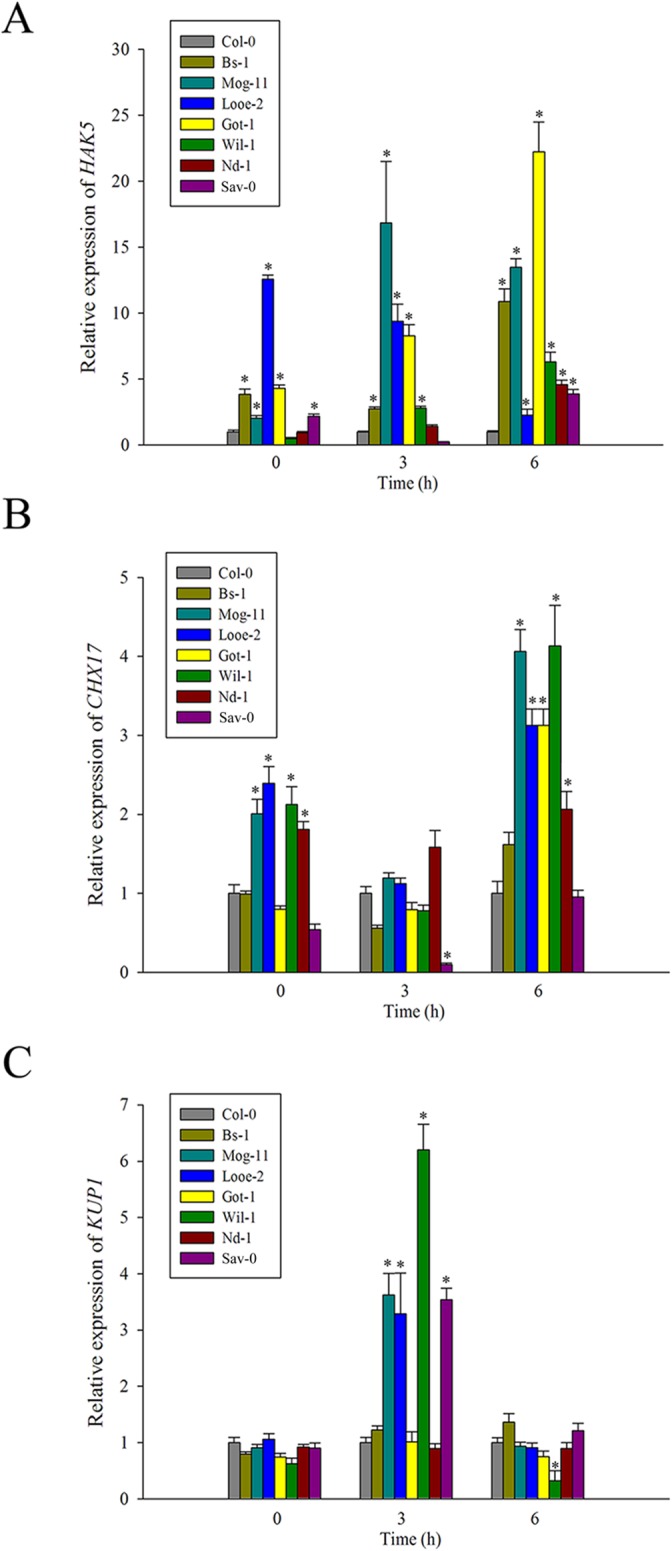
Expression analysis of genes related to K^+^ transport by qRT-PCR. The transcript levels of (A) *HAK5*, (B) *CHX17* and (C) *KUP1* in Col-0 and the selected accessions exposed to 100 mM NaCl for 0, 3 and 6 h. Values given as mean ± SE (*n* = 3). Significant differences from Col-0 accession at the same time point are indicated by * or ** (*P* < 0.05, *P* < 0.01).

Previously, some important K^+^ transporters such as HAK5, CHX17 and KUP1 were reported involved in K^+^ uptake and transport. Based on the high K^+^ content and low K^+^ efflux in the selected tolerant accessions, we tried to find that if the high K^+^ level were connected with the expression of the K^+^ transporters genes *AtHAK5*, *AtCHX17* and *AtKUP1*. When the transcription levels of them were analyzed by qRT-PCR, it became clear that *AtHAK5* was up-regulated in almost all tolerant accessions, especially in Mog-11 and Got-1, in which the *AtHAK5* expression level were increased more than 10-fold compared to that of Col-0 (Fig 3A and S4 Table). The abundance of *AtCHX17* transcript was also higher in the majority of the selections following a 6 h exposure to NaCl, especially in Mog-11 and Wil-1 (Fig 3B and S4 Table), whereas a similar response of *AtKUP1* appeared at 3 h exposure to NaCl, the peak was in that of Wil-1 (Fig 3C and S4 Table). The level of their high transcript corresponded to the high K^+^ level and low K^+^ efflux in all tolerant accessions in response to salinity stress (Fig 2B and Fig 2D), implying that these K^+^ transporters are active in improving the K^+^ retention in them. Together with the measurements of K^+^ content and K^+^ flux, it was also supported that salt tolerance correlated positively with a high ability to retain K^+^ in the selected tolerant accessions.

### Natural genetic variation and the transcription behavior of the *SOS* pathway genes

To examine whether those *Arabidopsis* variants for salt tolerance rely on polymorphism in the SOS genes, the coding regions of *AtSOS1*, *AtSOS2*, and *AtSOS3* were re-sequenced from the seven tolerant accessions (S2 Fig and S5 Fig). Two sequence variants in the AtSOS1 C terminal auto-inhibitory domain were observed among the tolerant plants’ alleles, compared to that of the Col-0 allele (S2 Fig); the T116A variant was present in six of the seven selections (the exception was Nd-1), and the D1125E variant was present in one (Looe-2) (S2 Fig). Both variants were also carried by the salinity sensitive accession Mh-1 (S2–S3 Fig), as well as by a number of other sequenced accessions at http://1001genomes.org/ (S4 Fig). The DSPS motif, which is phosphorylated by SOS2, was invariant (S2 Fig). The AtSOS2 sequence was identical to that in Col-0 for all the selections (S5 Fig). AtSOS3, the only polymorphism observed was V138I, present exclusively in Mog-11 (S5 Fig). Thus, there is little genetic variation in the sequence variation of the *SOS* genes among the tested *Arabidopsis* accessions.

**Fig 4:**
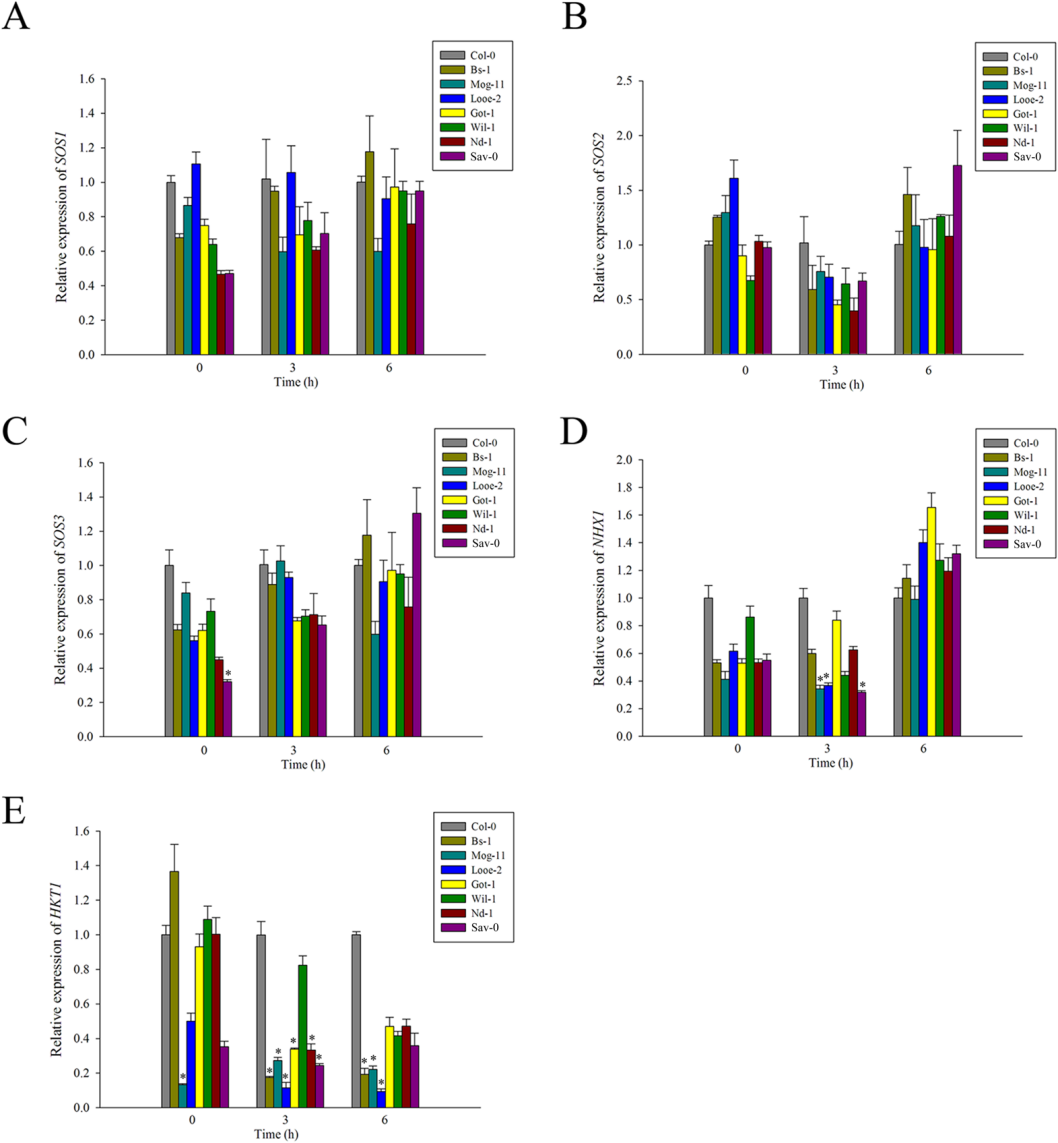
Expression analysis of genes related to Na^+^ transport by qRT-PCR. The transcript levels of (A) *SOS1*, (B) *SOS2*, (C) *SOS3*, (D) *NHX1* and (E) *HKT1;1* in Col-0 and the selected accessions exposed to 100 mM NaCl for 0, 3 and 6 h. Values given as mean ± SE (n = 3). Significant differences from Col-0 accession at the same time point are indicated by * (*P* < 0.05).

Furthermore, the re-sequencing was followed by an analysis of the *AtSOS* genes transcript abundance in salinity challenged plants. After both 3 and 6 h exposure to stress, the levels of *AtSOS1*, *AtSOS2* and *AtSOS3* transcript present were nearly similar and not high significantly in all seven entries to the respective ones present in Col-0 (Fig 4A and 4B and S5 Table), which could be in line with no significant difference in the Na^+^ contents of the selected accessions compared to that of Col-0 (Fig 2A). These data gave a hint that the SOS signaling pathway has highly conserved in *Arabidopsis* and play a fundamental role for the differential salinity tolerance among the selected accessions.

### Natural genetic variation and the transcription behavior of both *AtNHX1* and *AtHKT1;1*

Re-sequencing of the *AtNHX1* coding region especially for its C-terminal domain revealed no polymorphisms among the tolerant accessions (S5 Fig), nor was there a significant increase in the transcription level of *AtNHX1* in the tolerant selections compared to that of Col-0 with a exposure to NaCl treatment (Fig 4D and S5 Table). Moreover, re-sequencing of *AtHKT1;1* revealed two variants (R003I and L024V) only in Wil-1 (S5 Fig), and the abundance of *AtHKT1;1* transcript in response to NaCl stress was also not increased in most of the seven selected accessions compared to Col-0 (Fig 4E and S5 Table). Thus, there were little variation in the gene sequences and transcription behaviors of *AtNHX1* and *AtHKT1;1* in the tolerant selections. Alongside the lack of variation noted for Na^+^ content, it was concluded that Na^+^ seclusion or recirculation by AtNHX1 and AtHKT1;1 has also no positive relationship with differential salinity tolerance of the selected accessions.

### The accumulation of compatible solutes

**Fig 5:**
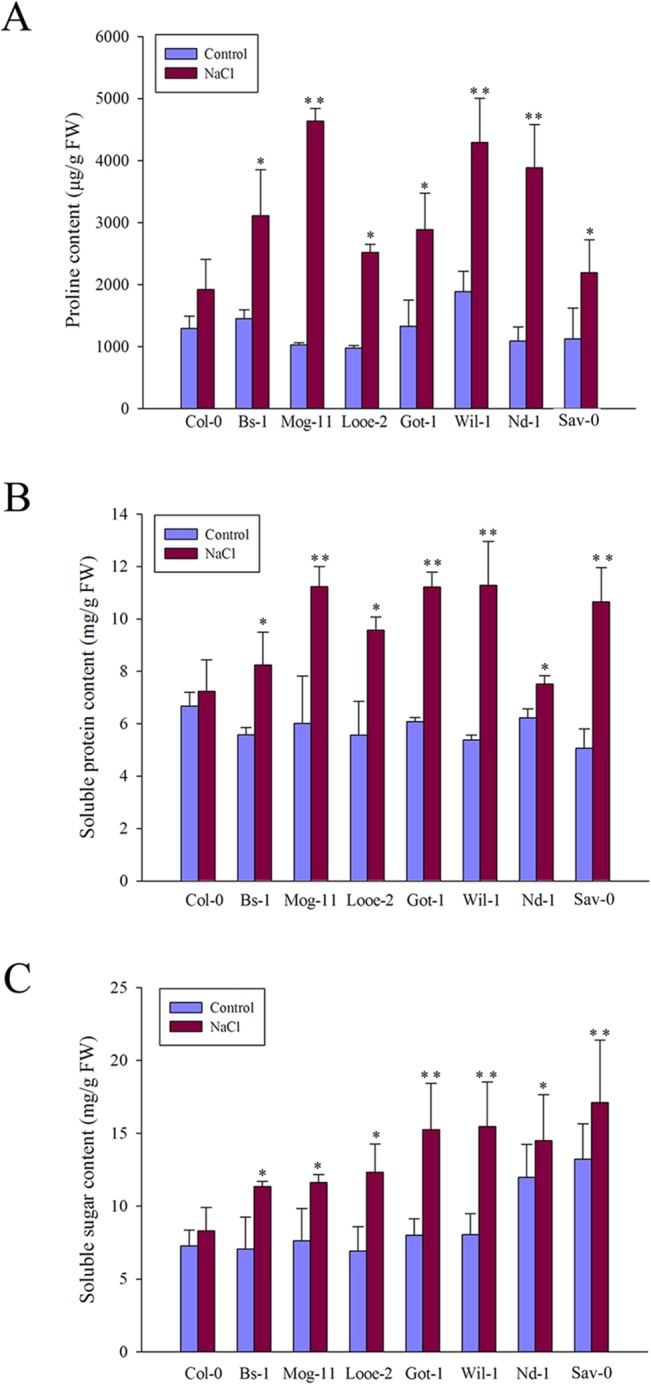
The contents of compatible solutes. (A) The content of proline in Col-0 and the selected accessions exposed to 0 or 200 mM NaCl for 36 h. (B) The contents of soluble proteins and (C) soluble sugar in Col-0 and the selected accessions exposed to 0 or 200 mM NaCl for 12 h. Values given as mean ± SE (*n* = 3), each replicate comprised at least 15 seedlings. Significant differences from the performance of Col-0 accession are indicated by * or ** (*P* < 0.05, *P* < 0.01).

Next, the differences of compatible solutes contents between the selected tolerant accessions and Col-0 were investigated. When plants exposed to 200 mM NaCl, the contents of compatible solutes including proline, soluble protein and soluble sugar were measured respectively. The results showed that the seven selected accessions were different from each other, but all had a higher levels of proline, soluble protein and soluble sugar than Col-0 (Fig 5 and S8 Fig), which were associated differently with the NaCl-induced net K^+^ flux in each other (S6 Table).

**Fig 6:**
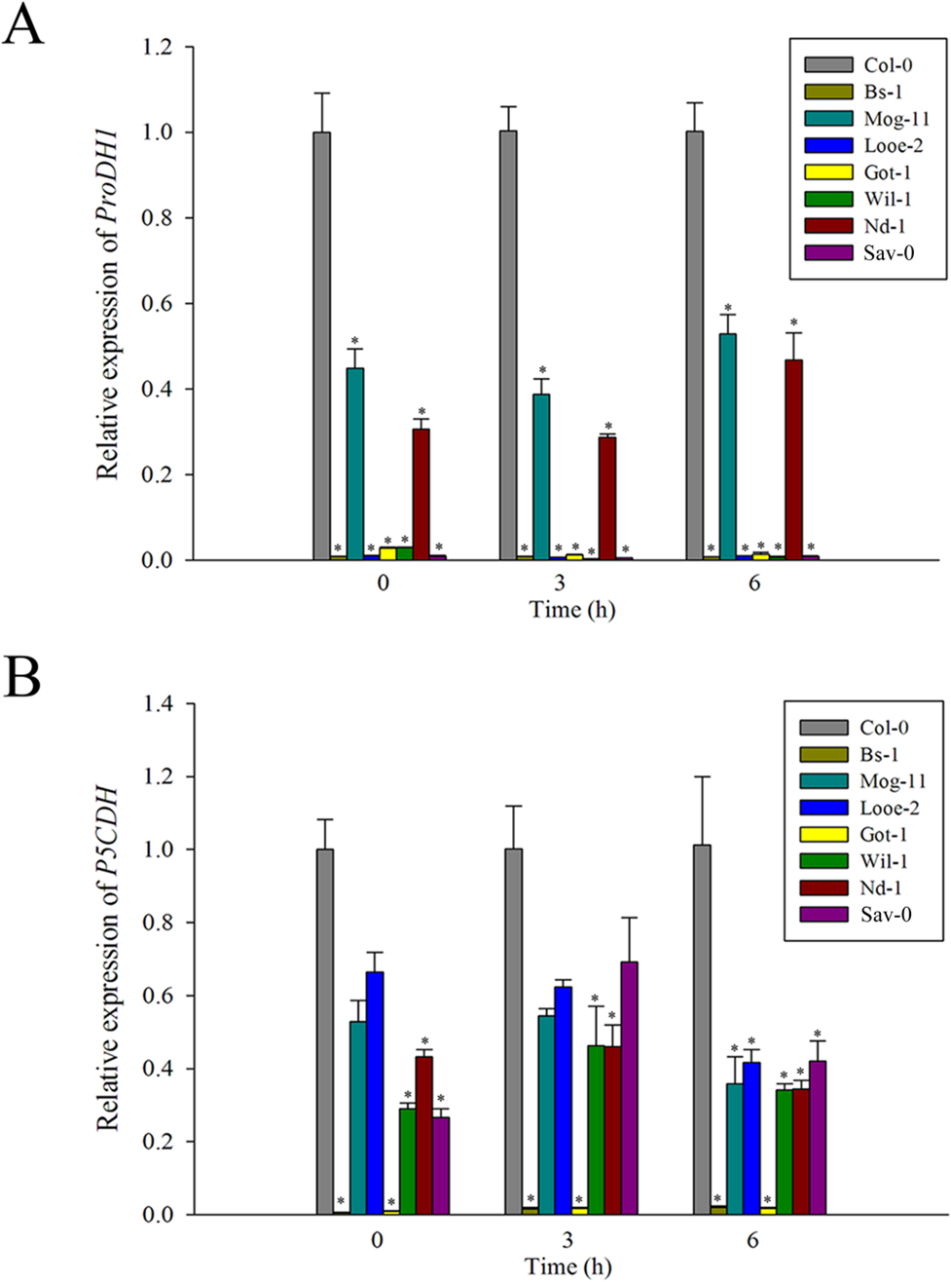
Expression analysis of genes related to proline degradation by qRT-PCR. The transcript levels of (A) *ProDH1* and (B) *P5CDH* in Col-0 and the selected accessions exposed to 100 mM NaCl for 0, 3 and 6 h. Values given as mean ± SE (*n* = 3). Significant differences from Col-0 accession at the same time point are indicated by * (*P* < 0.05).

As for proline, the transcript abundance of *AtProDH1* and *AtP5CDH*, which encode enzymes involved in the proline degradation pathway [81], was analyzed via qRT-PCR. The results showed that *AtProDH1* and *AtP5CDH* were less strongly transcribed in the selected tolerant accessions than in Col-0, and nearly the same difference was observed in non-stressed plants (Fig 6), suggesting that low levels of both *AtProDH1* and *AtP5CDH* expression were the main cause of higher proline levels in the tolerant accessions (Fig 5A). In addition, *AtP5CR*, *AtP5CS1* and *AtP5CS2* which encode enzymes involved in the proline biosynthsis pathway [81], were also analyzed via qRT-PCR at 3 and 6 h after salt treatment. However, these genes regulating proline biosynthesis were not changed positively compared to that of Col-0, except Looe-2 at 3 h after salt stress (S6 Fig). These results suggested that the higher proline content is correlated closely with the decreased *AtProDH1* and *AtP5CDH* expression in the selected tolerant accessions.

### Redox response after salt treatment

**Fig 7:**
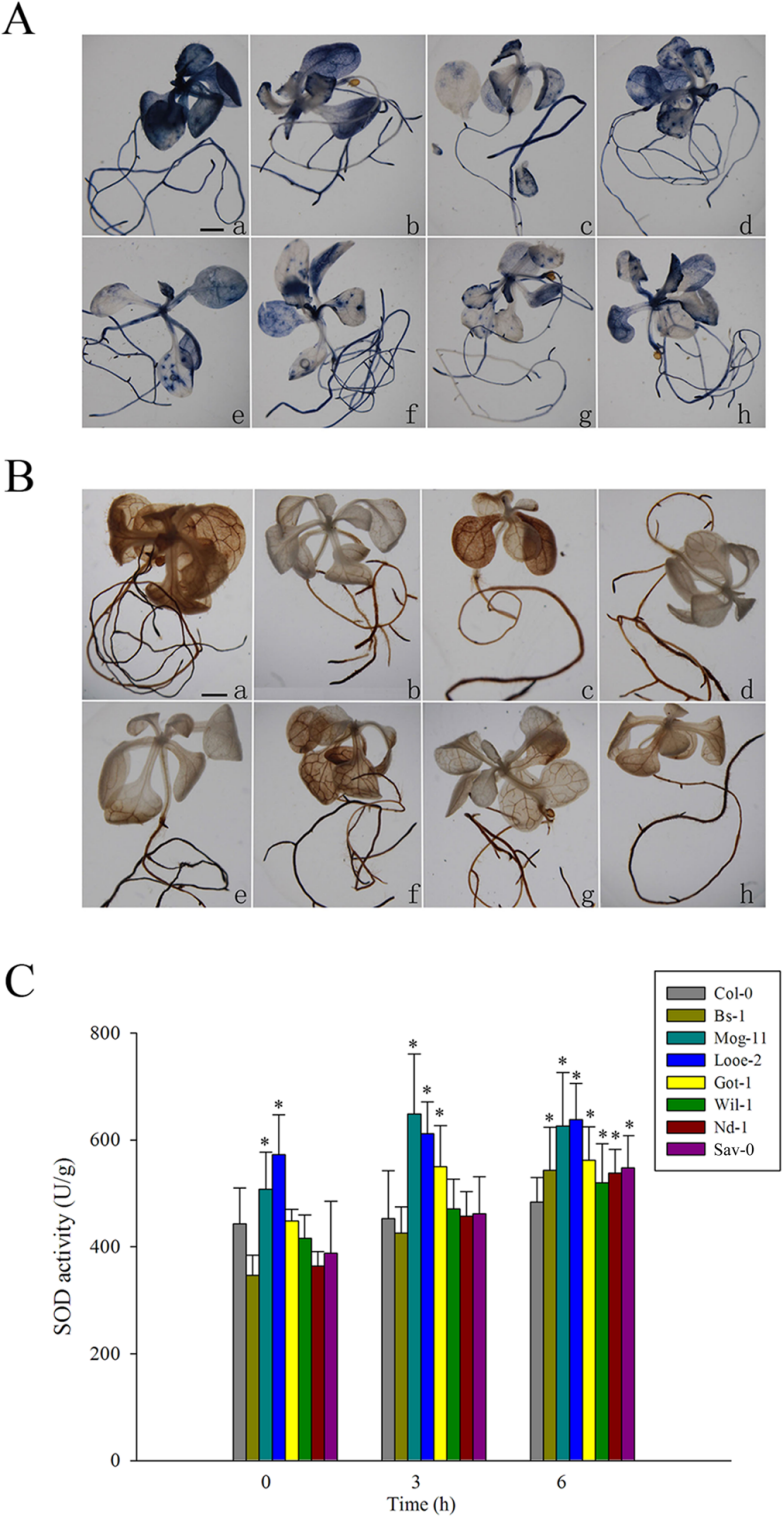
Accumulation of oxidants and superoxide dismutase (SOD) activity in response to salinity stress. (A) Superoxide radical, detected by nitroblue tetrazolium (NBT) staining, (B) H_2_O_2_, detected by 3, 3’-diaminobenzidine (DAB) staining in Col-0 and the selected accessions exposed to 150 mM NaCl for 10 days. a: Col-0; b: Bs-1; c: Mog-11; d: Looe-2; e: Got-1; f: Wil-1; g: Nd-1; h: Sav-0. Bar = 0.1 cm. (C) SOD activity in Col-0 and the selected accessions exposed to 100 mM NaCl for 0, 3 and 9 h. Values given as mean ± SE (*n* = 3), each replicate comprised at least 15 seedlings. Significant differences from the performance of Col-0 accession at the same time point are indicated by * (*P* < 0.05).

In order to examine whether there are difference in ROS levels between the tolerant selections and Col-0 under salinity stress, NBT staining and DAB staining were carried out to assess the ROS contents. The results showed that salinity treatment promoted the accumulation of the superoxide radical and hydrogen peroxide, but to a lesser extent in the seven selected accessions than in Col-0 (Fig 7A and 7B). Moreover, the SOD activity was responsive to the stress, after a 3 or 9 h exposure to stress, there was a greater level of enzyme activity in the tolerant selections than in Col-0 (Fig 7C).

**Fig 8:**
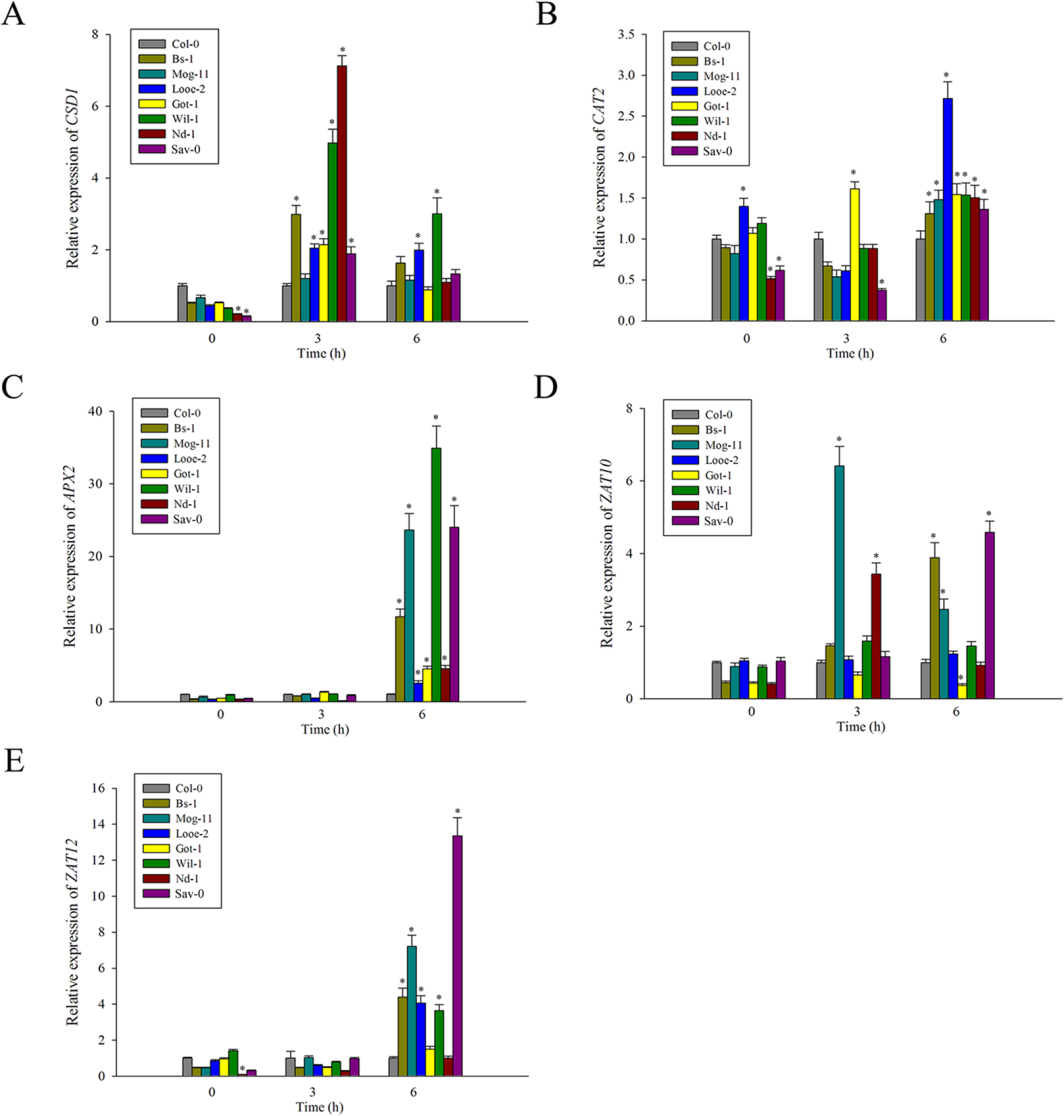
Expression analysis of genes related to ROS metabolism by qRT-PCR. The transcript levels of (A) *CSD1*, (B) *CAT2*, (C) *APX2*, (D) *ZAT10* and (E) *ZAT12* in Col-0 and the selected accessions exposed to 100 mM NaCl for 0, 3 and 6 h. Significant differences from Col-0 accession at the same time point are indicated by * (*P* < 0.05).

On the other hand, the transcription levels of *AtCSD1*, *AtCAT2*, *AtAPX2* respectively encoding SOD, catalase (CAT) and ascorbate peroxidase (APX), as well as two transcription factors *AtZAT10* and *AtZAT12* participating in the ROS signaling pathways were several-times higher in most of the selections than in Col-0 (Fig 8). Interestingly, the high expression levels of *AtAPX2* emerged remarkably in Mog-11, Wil-1 and Sav-0, more than 20-fold change relative to that of Col-0 (Fig 8C). In addition, *AtCSD1*, as one of three Cu/Zn SOD genes, its expression were higher in almost all the selections (except Mog-11) (Fig 8A), which could be correlated with their higher SOD activity compared to that of Col-0 (Fig 7C). Thus, these results demonstrated that the lower ROS amounts in the detected tolerant accessions could be attributed to their higher SOD activity together with the elevated transcription levels of those relevant genes in response to salinity stress.

## Discussion

*Arabidopsis* accessions are highly variable in genotypes and phenotypes [82]. In the present study, a screen of 82 accessions, collected from diverse habitats, was carried out to investigate the extent of variation in the response to salinity stress (S1 Table), resulting in the identification of seven accessions noticeably more tolerant of salinity than the reference accession Col-0 (Fig 1A). The tolerance of the selected seven accessions was manifested by a better ability to maintain root growth (Fig 1B), a weaker tendency for the cotyledons to become bleached (Fig 1C) and higher chlorophyll content than that of Col-0 (Fig 1D). Moreover, the selections had less MDA accumulation under salt stress, indicated decreased cell membrane damage compared to Col-0 (Fig 1E). Therefore, these results indicated that the seven accessions tolerate salinity better than Col-0 (Fig 1 and S1 Table). Previously, Ts-1 and Tsu-1, directly located on the coastal regions, were selected as two salt tolerant accessions [68]. In the present study, we also screened them and found that these two accessions were more tolerant than Col-0, but less than the selected seven accessions evaluated by an assay using agar plates. The different results in salt tolerance probably due to the two different assays for the plants grown on the two media, the similar case also reported previously [56, 83]. However, in this study, the improved tolerance of the selections was in agreement with the evaluation by using soil or hydroponics assay (S1 Fig).

It has been found that hypersensitivity to salt stress was frequently associated with poor K^+^ absorption in *Arabidopsis* and tomato mutants [38, 84], and the maintenance of high K^+^ levels and a low Na^+^/K^+^ ratio in the cytoplasm could also be essential for salt tolerance [85–86]. This is consistent with our results, when exposed to salinity stress, the seven tolerant *Arabidopsis* selections had higher K^+^ contents, maintained a lower Na^+^/K^+^ ratio than that of the standard accession Col-0 (Fig 2B and 2C), suggesting that the maintenance of higher K^+^ contents in the tolerant selections contributed significantly to salinity tolerance of them. Further to measure the net K^+^ flux among them, it was interestingly found that K^+^ efflux for all the tested accessions even that in normal condition, but the salt-tolerant selections showed less K^+^ efflux than Col-0 (Fig 2D), suggesting that the higher K^+^ content in the selections was attributed to the decreased K^+^ efflux in them. Thus, the tolerant ecotypes possessed the strong ability to retain K^+^ or prevent K^+^ loss under salt stress. In fact, the positive relationship between K^+^ retention and salinity tolerance was also supported by increasing similar findings in other species such as barley, wheat and *Thellungiella halophila* [39–41, 87]. Some high-affinity K^+^ transporters including HAK5, CHX17 and KUP1 were previously reported to play active roles for K^+^ uptake, accumulation and homeostasis under a low K^+^ condition [47, 49, 52–53, 88–89]. Consistently, in this study, a comparative analysis of *AtHAK5*, *AtCHX17* and *AtKUP1* expression levels indicated that there is a positive relationship the correlation between these genes and high K^+^ content in the selected accessions. As such, *AtHAK5* transcript abundance in the tolerant accessions was significantly higher than in Col-0, most markedly in Mog-11 and Got-1 after salt treatment (Fig 3A and S4 Table). *AtCHX17* and *AtKUP1* were both inducible obviously by salinity in different treated time point (Fig 3B and 3C and S4 Table), especially in the tolerant accessions including Mog-11, Looe-2 and Wil-1 (Fig 3B and 3C). The higher increased expression of *AtHAK5*, *AtCHX17* and *AtKUP1* after salt treatment is consistent with the high ability to retain K^+^ in the tolerant accessions, implying that AtHAK5, AtCHX17 and AtKUP1 may be essential to improve K^+^ uptake and results in more K^+^ to be retained in the tolerant accessions. Next, it could be interesting to further explore whether the up-regulation of these genes is caused by the polymorphisms of them. Moreover, a strong K^+^ retention is also able attributed to the smaller salt-induced K^+^ leakage in the control of K^+^ channels including some of Shaker-like outward-rectifying depolarization-activated K^+^ channels, K^+^-Ca^2+^ activated outward-rectifying channels (KCO) and nonselective cation channels (NSCCs) [37, 90–93], and these K^+^ channels could also work together with the above K^+^ transporters to retain K^+^ or prevent K^+^ loss under salt stress. Previously, K^+^ efflux from plant tissues in *Arabidopsis* was largely conferred by the gated outward-rectifying depolarization-activated K^+^ channel (GORK) [94], and the *AtGORK* expression levels in the selections were also detected in this study, but no significant difference were found among them in response to salinity (S7 Fig). Over all, it was concluded that the strong K^+^ retention in the tolerant selections was possibly attributed to the high-efficient K^+^ uptake mediated actively by the AtHAK5, AtCHX17 and AtKUP1 transporter, rather than the smaller K^+^ leakage regulated by the AtGORK channel.

Salinity tolerance has also commonly been associated with an enhanced ability of Na^+^ detoxification and thereby to maintain a low Na^+^ in cytoplasm [2, 6–7, 26, 95]. The SOS signaling pathway and *AtNHX1*, two major factors were reported to export Na^+^ out of the cell or sequester Na^+^ within the vacuole, and *AtHKT1;1* are essential for root-to-shoot Na^+^ partitioning, also as a major player in plant salt tolerance [5–6, 16, 25, 31, 96]. Moreover, the critical functional region of the SOS1 protein is its auto-inhibitory C terminal SOS1 domain, which must be phosphorylated for the activation of the whole protein by the SOS2-SOS3 complex [97]. In the present study, the sequence comparison among the *Arabidopsis* accessions focused on this region identified that all but one carried the alternative allele to Col-0 at position 1116, which lies within the C terminal auto-inhibitory domain (S2 Fig). However, this allele cannot be critical for gene function, as it is also carried by both tolerant and sensitive accessions (S2–S4 Fig). Similarly, there were also no critical variations in the *AtSOS2*, *AtSOS3*, *AtNHX1* and *AtHKT1;1* of the selected accessions (S5 Fig). Moreover, there were little variations in the transcriptional behavior of the *AtSOS* genes, *AtNHX1* and *AtHKT1;1* genes (Fig 4 and S5 Table), which could be due to the polymorphism of the promoter regions of those genes among the selections. However, when challenged by NaCl, the transcription levels of *AtSOS* genes in the tolerant selections showed no significant changes (Fig 4A–4C and S5 Table), thus, it was concluded that there was no difference in the efficiency of regulating Na^+^ efflux at cellular level among the tested accessions, which correspond to the similar high Na^+^ levels of the tested accessions. Consistently, a similar transcriptional variation in response to salt stress in *Arabidopsis* were reported previously [71]. In addition, the transcription level of *AtNHX1* decreased in Mog-11, Looe-2 and Sav-0 at 3 h after salt treatment (Fig 4D and S5 Table), and it was still not clearly why the *AtHKT1;1* gene was expressed obviously lower in most of the tolerant ones than in Col-0 when exposed to salt stress (Fig 4E and S5 Table). In all, these results supported that the SOS signaling pathway, as well as AtNHX1 and AtHKT1;1, could be as a fundamental and conserved mechanism to the elevated salinity tolerance displayed by the seven accessions, but not a key special factor to distinct them from Col-0.

Salinity damage can be mitigated if the plant tissue effectively accumulates compatible solutes. The presence of these compounds relieves the osmotic pressure imposed by saline soil water and thus allows the plant to continue taking up water, stabilize the photosystem II complex, protect the structure of enzymes and proteins, maintain membrane integrity and protect cells against oxidative damage by scavenging ROS [6, 98–100]. In this study, an elevated level of such solutes, in particular that of proline, was found and could be as a feature of the tolerant accessions (Fig 5, S8 Fig and S5 Table), and consistent with this physiological difference, the lower transcription kinetics of *AtProDH1* and *AtP5CDH* which encode enzymes involved in proline degradation [81], were noted between the tolerant accessions and Col-0 when exposed to salinity stress (Fig 5A and Fig 6). A similar conclusion was previously reached for the salinity tolerant accession Bu-5 [56]. However, it was not observed a positive salinity response of some key enzyme genes involved in proline biosynthesis (S6 Fig), for which the reasonable explanations are possibly that de novo proline comes at high carbon cost and is not preferentially adopted by plants [81]. On the other hand, ROS play a dual role in plants acting as both key regulators of many biological processes including growth and development, cell cycle, hormone signaling, and as toxic by-products that cause oxidative stress and cell damage during different stress conditions [101–104]. Salinity stress has long been known to cause the over-accumulation of ROS in plants [105–106], and the excessive ROS scavenging is also regarded as a basic strategy for plant adaptation to stress [2, 5–6, 107]. Previously, it has been reported that higher proline levels might be essential to detoxify salt-tress induced ROS produced in plant tissues [100, 108–112], and high K^+^ content would also help to reduce the ROS amount in plants [113]. As a consequence, in this study, the tolerant accessions indeed exhibited a lower level of ROS than Col-0 (Fig 7A and 7B). Furthermore, it has already been reported that a number of enzymatic and non-enzymatic mechanisms to detoxify ROS existed in plant cells, such as SODs, which dismutate O_2_^·-^ to O_2_ and H_2_O_2_, is a key element of the enzymatic protective system [107, 114], and over-expression of various SOD genes in higher plants enhanced tolerance to salinity stress [115]. Consistently, we also found a higher SOD activity in the tolerant accessions compared to Col-0, and a higher transcript level of *AtCSD1* (except Mog-11) (Fig 7C and Fig 8A). In addition, there were also higher transcript abundance of *AtCAT2* and *AtAPX2* (Fig 8B and 8C), suggesting that these protective enzymes participated actively in ROS scavenging. Notably, APX has a higher affinity for H_2_O_2_ than CAT, and could play a more active role in ROS scavenging by according to their elevated expression of *AtAPX2*, especially in Mog-11, Wil-1 and Sav-0 (Fig 8C). Moreover, two upstream transcription factors *AtZAT10* and *AtZAT12* in the ‘ROS gene network’ [2, 102], were also up-regulated significantly in the tolerant accessions (Fig 8D and 8E), and the data was in agreement with the previous report [71]. Therefore, we concluded that the tolerant accessions also possessed these adaptive mechanisms of slowing down proline degradation and improving ROS scavengers to enhance salinity tolerance.

In conclusion, several salt-tolerant accessions have been identified from a collection of over 80 diverse accessions, while their differential salt tolerance is independent to the essential *SOS* pathway and other well-known Na^+^-related transporter genes, as *AtNHX1* and *AtHKT1;1*, as well as the Na^+^ content in plants, However, the high K^+^ content, which is the result of the low K^+^ efflux and high expression of the K^+^ transporters genes *AtHAK5*, *AtKUP1* and *AtCHX17*, plays important role in the salinity response of these tolerant accessions. An elevated level of compatible solutes and a reduced level of ROS in plants provided further physiological indicators of the tolerant plants’ improved ability to adapt to the salinity stress. These data suggest that the tolerant accessions were better pre-conditioned to survive salinity stress through more responsive regulation of K^+^ homeostasis and more sensitive transcription of key stress response genes.

## Supporting Information

S1 Fig The response of Col-0 and the selected accessions to salinity stress. (A) Soil-grown 3-week-old plants watered with 0 or 500 mM NaCl for 20 days, (B) Hydroponics-grown 5-day-old seedlings exposed to 0 or 200 mM NaCl for 3 days. a: Col-0; b: Looe-2; c: Mog-11; d: Wil-1; e: Nd-1; f: Bs-1; g: Sav-0; h: Got-1. Col-0 is marked by a red ellipse.
(TIF)

S2 Fig Polymorphism in the SOS1 peptide sequence. (A) The structure of SOS1. The transmembrane domain is shown in yellow, the cytosolic domain in blue, the cyclic nucleotide binding domain in green and the autoinhibitory domain in red. (B) Allelic variation for the SOS1 sequence among the selected accessions. SOS1 sequences of the indicated species were aligned using CLUSTAL-W. The SOS1 sequence of the salinity-sensitive accession Mh-1 is shown in S3 Fig Peptide changes are marked by vertical red lines and solid arrowheads (blue arrowhead: T1116A, grey arrowhead: D1125E). The DSPS motif is boxed in red.
(TIF)

S3 Fig The response of the salinity-sensitive accession Mh-1 to salinity stress. The 4-day-old seedlings of Mh-1 and Col-0 accessions were exposed to 0 or 150 mM NaCl for 15 days. Bar = 0.5 cm.
(TIF)

S4 Fig Allelic variation for the *SOS1* sequence among other sequenced accessions revealed from http://1001genomes.org/. *SOS1* sequences of the indicated species were aligned using CLUSTAL-W. Peptide changes are marked by blue arrowhead: T1116A.
(TIF)

S5 Fig Polymorphism in *SOS1*, *SOS2*, SOS3, *NHX1* and *HKT1;1*. Domain structure of (A) *SOS1* (see also S2 Fig), (B) *SOS2*, (C) *SOS3*, (D) *NHX1* and (E) *HKT1;1*. The SOS2 FISL/NAF motif is displayed in cyan, the protein phosphatase interaction (PPI) motif in blue, and the rest of PPI domain in green. The four SOS3 EF-hand motifs are displayed in orange, and the vertical red line indicates variable residue V138I in EF-hand 3. NHX1 contains 12 transmembrane motifs and HKT1;1 eight. The vertical red lines in HKT1;1 represent variable residues R003I and L024V.
(TIF)

S6 Fig Expression analysis of genes related to proline biosynthesis by qRT-PCR. The transcript levels of (A) *P5CR*, (B) *P5CS1* and (C) *P5CS2* in Col-0 and the selected accessions exposed to 100 mM NaCl for 0, 3 and 6 h. Values given as mean ± SE (*n* = 3). Significant differences from Col-0 accession at the same time point are indicated by * (*P* < 0.05).
(TIF)

S7 Fig Expression analysis of *GORK* gene by qRT-PCR. The transcript level of *GORK* in Col-0 and the selected accessions exposed to 100 mM NaCl for 0, 3 and 6 h. Values given as mean ± SE (*n* = 3), Significant differences from Col-0 accession at the same time point are indicated by * (*P* < 0.05).
(TIF)

S8 Fig Fold changes of compatible solutes in the selected tolerant accessions relative to that of Col-0 under 200 mM NaCl stress. (TIF)

S1 Table Origin and responses to salt stresses of 82 *Arabidopsis thaliana* accessions. (DOC)

S2 Table Primers used for genome sequence analysis. (DOC)

S3 Table Primers used for real-time PCR analyses. (DOC)

S4 Table Expression profile of *AtHAK5*, *AtCHX17*, *AtKUP1* and *AtGORK* gene. The expression levels of these genes normalized to *ACTIN2* gene respectively were analyzed in Col-0 and the selected tolerant accessions exposed to 100 mM NaCl for 0, 3 and 6 h. Values given as mean ± SE (*n* = 3).
(DOC)

S5 Table Expression profile of *AtSOS1*, *AtSOS2*, *AtSOS3*, *AtNHX1* and AtHKT1;1 gene. The expression levels of these genes normalized to *ACTIN2* gene respectively were analyzed in Col-0 and the selected tolerant accessions exposed to 100 mM NaCl for 0, 3 and 6 h. Values given as mean ± SE (*n* = 3).
(DOC)

S6 Table Linear correlation (*r* ^*2*^) between NaCl-induced net K^+^ flux (100 mM NaCl) and the content of compatible solutes determined under 200 mM NaCl stress in this study. (DOC)
