# Supplementary figures and images for: Potassium Retention under Salt Stress Is Associated with Natural Variation in Salinity Tolerance among Arabidopsis Accessions
Source: PLoS One. 2015 May 19;10(5):e0124032. doi: 10.1371/journal.pone.0124032 (PMC4438003; doi:10.1371/journal.pone.0124032)

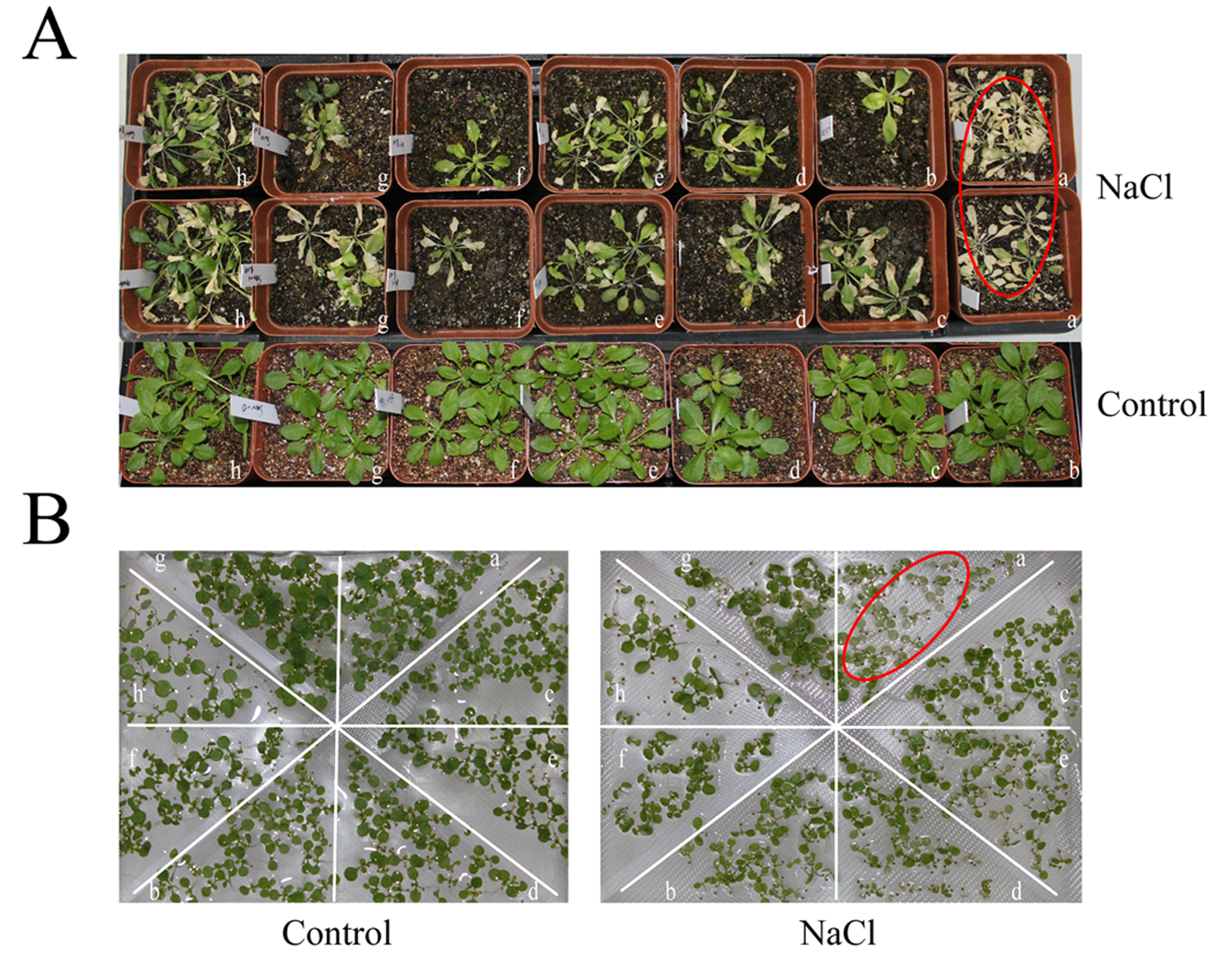

Supplement: S1 Fig — (A) Soil-grown 3-week-old plants watered with 0 or 500 mM NaCl for 20 days, (B) Hydroponics-grown 5-day-old seedlings exposed to 0 or 200 mM NaCl for 3 days. a: Col-0; b: Looe-2; c: Mog-11; d: Wil-1; e: Nd-1; f: Bs-1; g: Sav-0; h: Got-1. Col-0 is marked by a red ellipse. (TIF) [file pone.0124032.s001.tif]

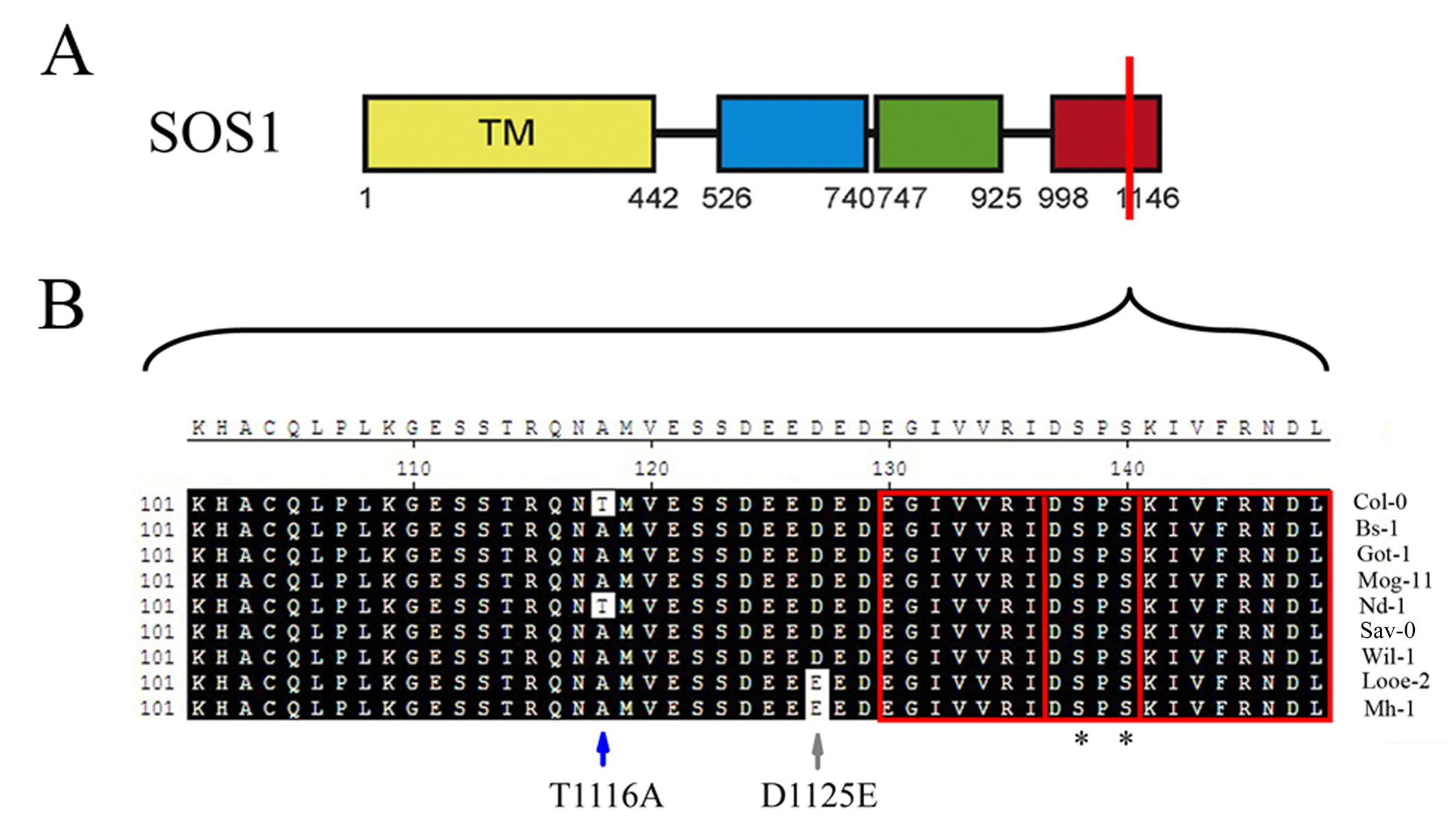

Supplement: S2 Fig — (A) The structure of SOS1. The transmembrane domain is shown in yellow, the cytosolic domain in blue, the cyclic nucleotide binding domain in green and the autoinhibitory domain in red. (B) Allelic variation for the SOS1 sequence among the selected accessions. SOS1 sequences of the indicated species were aligned using CLUSTAL-W. The SOS1 sequence of the salinity-sensitive accession Mh-1 is shown in S3 Fig Peptide changes are marked by vertical red lines and solid arrowheads (blue arrowhead: T1116A, grey arrowhead: D1125E). The DSPS motif is boxed in red. (TIF) [file pone.0124032.s002.tif]

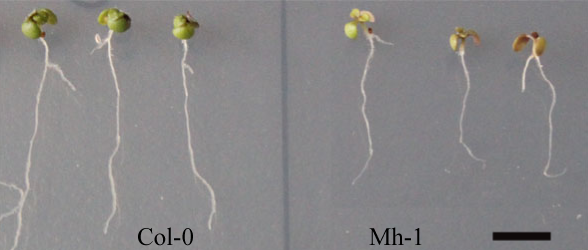

Supplement: S3 Fig — The 4-day-old seedlings of Mh-1 and Col-0 accessions were exposed to 0 or 150 mM NaCl for 15 days. Bar = 0.5 cm. (TIF) [file pone.0124032.s003.tif]

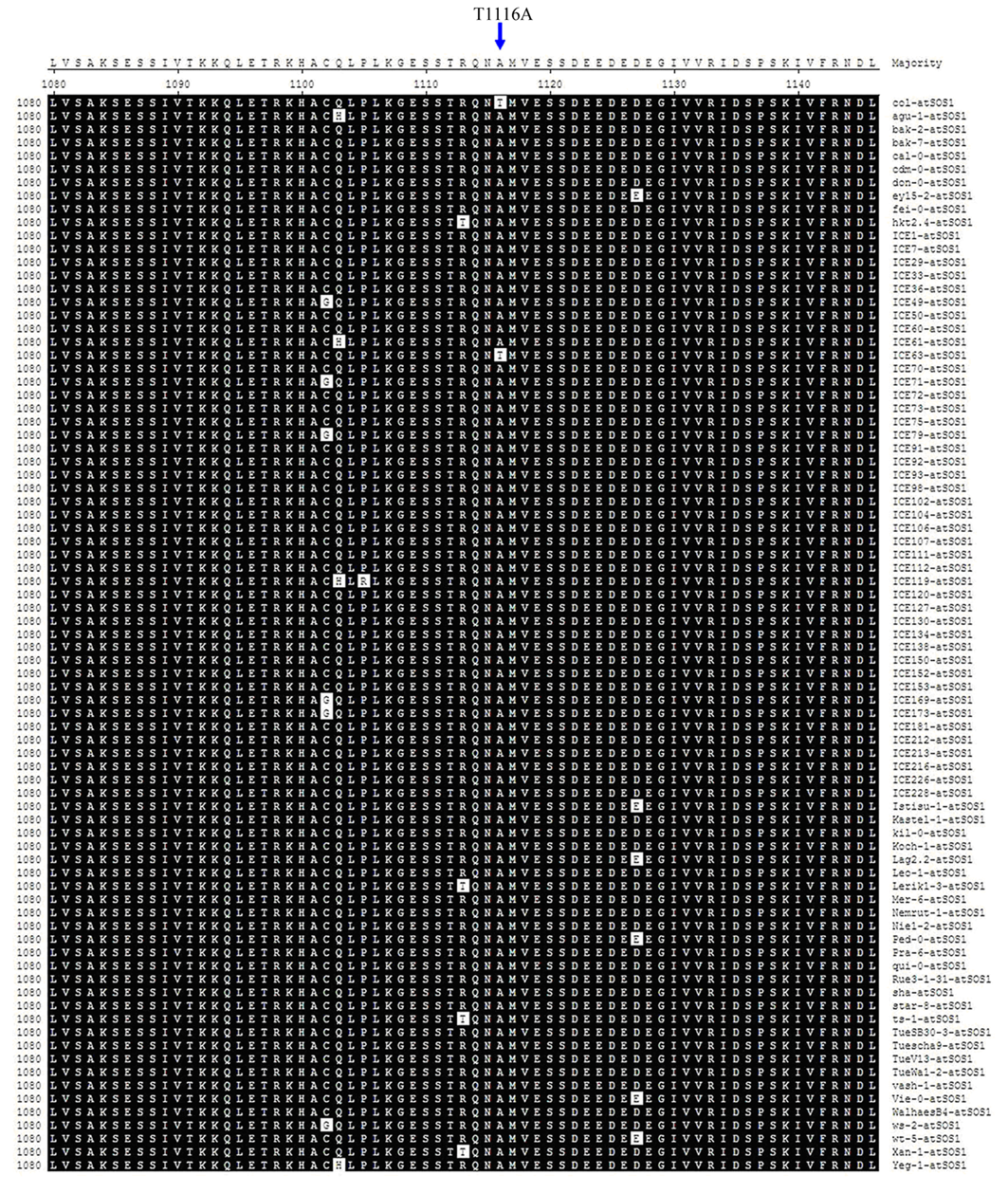

Supplement: S4 Fig — SOS1 sequences of the indicated species were aligned using CLUSTAL-W. Peptide changes are marked by blue arrowhead: T1116A. (TIF) [file pone.0124032.s004.tif]

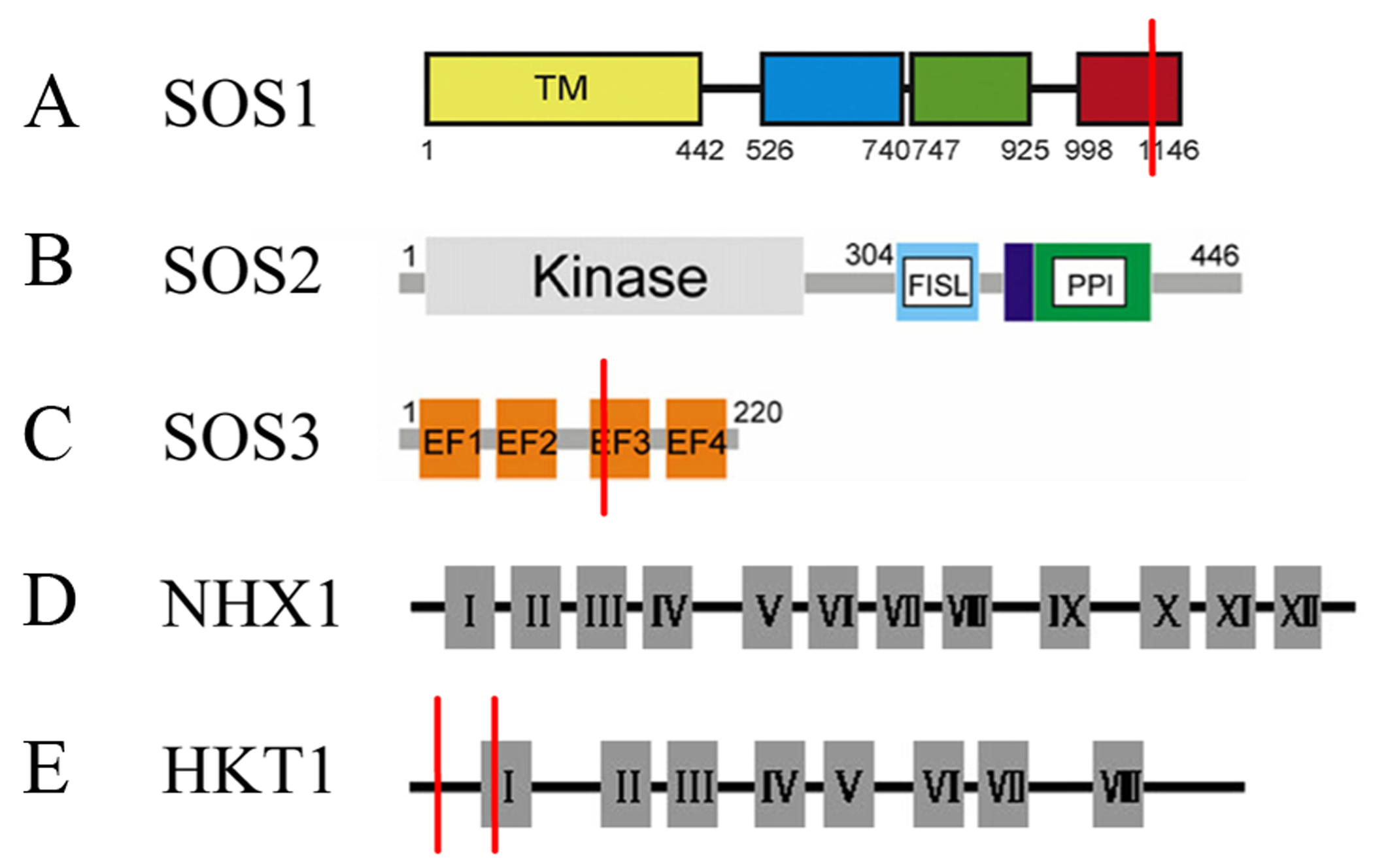

Supplement: S5 Fig — Domain structure of (A) SOS1 (see also S2 Fig), (B) SOS2, (C) SOS3, (D) NHX1 and (E) HKT1;1. The SOS2 FISL/NAF motif is displayed in cyan, the protein phosphatase interaction (PPI) motif in blue, and the rest of PPI domain in green. The four SOS3 EF-hand motifs are displayed in orange, and the vertical red line indicates variable residue V138I in EF-hand 3. NHX1 contains 12 transmembrane motifs and HKT1;1 eight. The vertical red lines in HKT1;1 represent variable residues R003I and L024V. (TIF) [file pone.0124032.s005.tif]

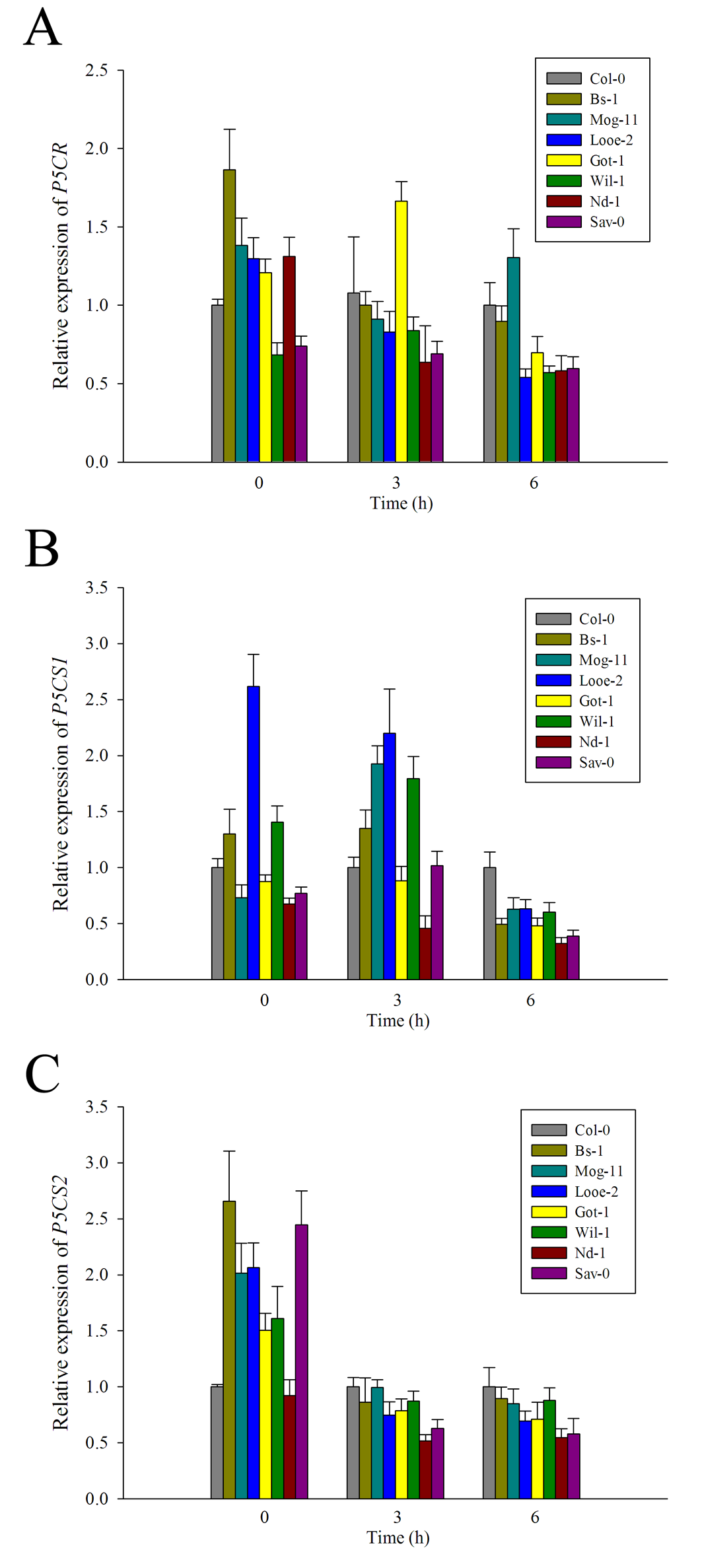

Supplement: S6 Fig — The transcript levels of (A) P5CR, (B) P5CS1 and (C) P5CS2 in Col-0 and the selected accessions exposed to 100 mM NaCl for 0, 3 and 6 h. Values given as mean ± SE (n = 3). Significant differences from Col-0 accession at the same time point are indicated by * (P < 0.05). (TIF) [file pone.0124032.s006.tif]

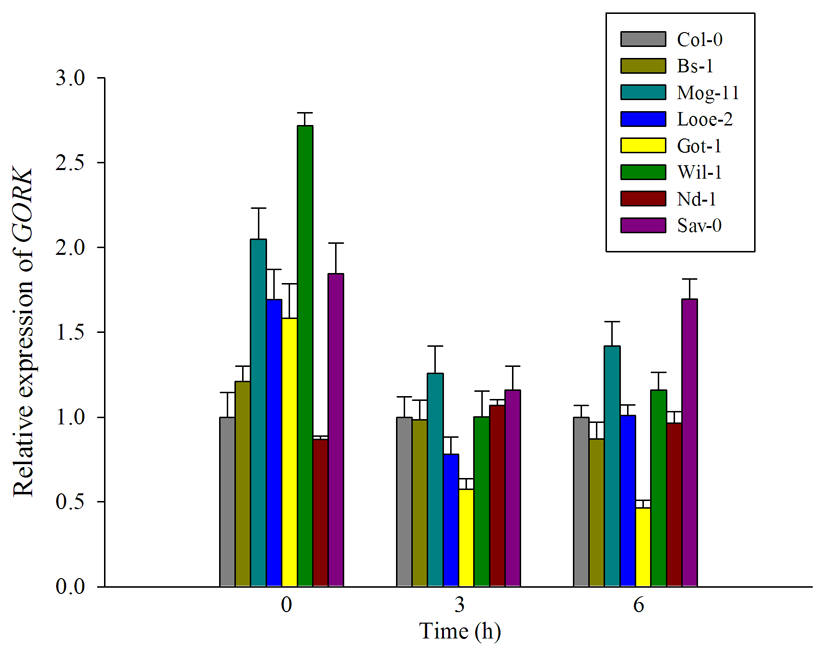

Supplement: S7 Fig — The transcript level of GORK in Col-0 and the selected accessions exposed to 100 mM NaCl for 0, 3 and 6 h. Values given as mean ± SE (n = 3), Significant differences from Col-0 accession at the same time point are indicated by * (P < 0.05). (TIF) [file pone.0124032.s007.tif]

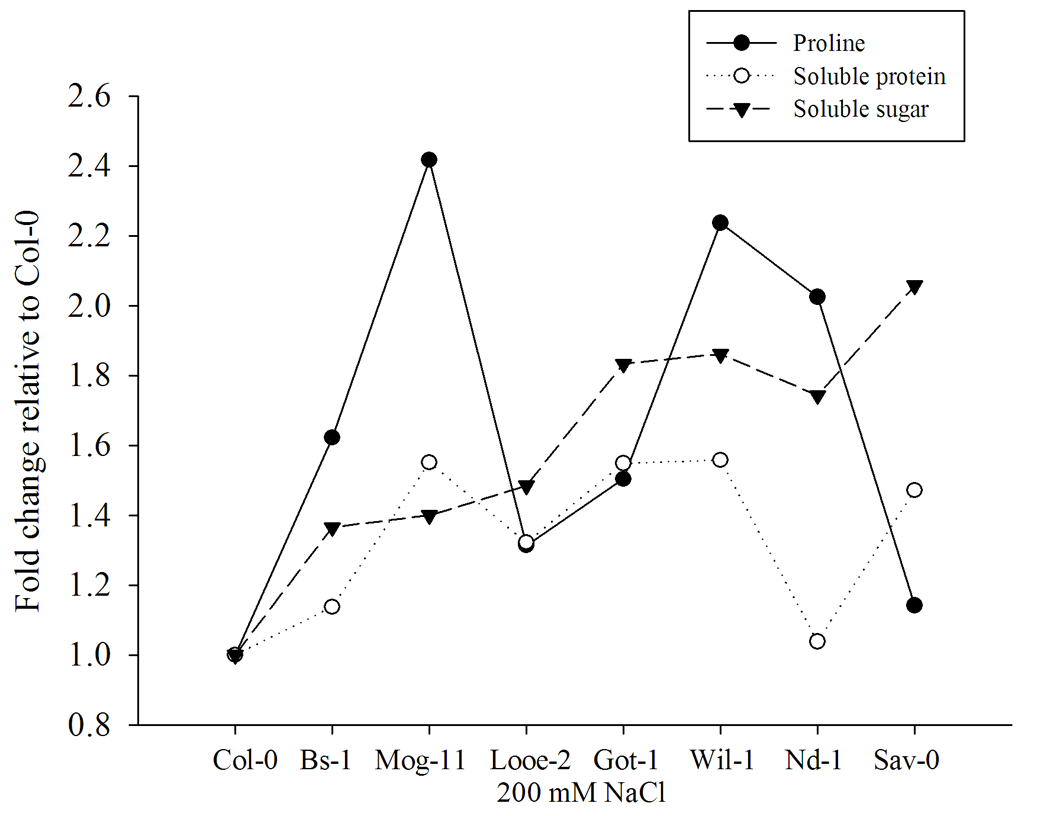

Supplement: S8 Fig — (TIF) [file pone.0124032.s008.tif]
