# Supplementary material for: Potassium Retention under Salt Stress Is Associated with Natural Variation in Salinity Tolerance among Arabidopsis Accessions
Source: PLoS One. 2015 May 19;10(5):e0124032. doi: 10.1371/journal.pone.0124032 (PMC4438003; doi:10.1371/journal.pone.0124032)
Supplement: S1 Table — (DOC) [file pone.0124032.s009.doc]

**S1**_**Table.doc Origin and responses to salt stresses of 82 *Arabidopsis thaliana* accessions.**

| Accession | Country | Stock Number | Relative primary root length (%, 150 mM NaCl) | Cotyledon albino rate (%, 200 mM NaCl) |
| --- | --- | --- | --- | --- |
| Mh-1 | Poland | CS6793 | 0.0304 | 1.0000 |
| Cnt | UK | CS1635 | 0.0434 | 0.8333 |
| Sf-2 | Spain | CS1516 | 0.0529 | 0.2857 |
| Pa-1 | Italy | N1439 | 0.0585 | 0.3333 |
| Esp-1 II | Norway | CS40021 | 0.0623 | 0.9600 |
| Ka-0 | Austria | [CS6752](http://www.arabidopsis.org/servlets/TairObject?type=stock&id=204291) | 0.0628 | 0.8000 |
| Ws-2 | Russia | CS2360 | 0.0663 | 0.9750 |
| Ei-2 | Germany | CS6689 | 0.0676 | 0.8750 |
| Lac-2 | France | W40288 | 0.0764 | 1.0000 |
| Seat-1 |  | CS22341 | 0.0793 | 0.5000 |
| Terlet-1 | Netherlands | CS75950 | 0.0804 | 0.1818 |
| Fi-1 | Germany | CS6705 | 0.0839 | 0.8750 |
| Everlaan voor |  | W10170 | 0.0843 | 0.3000 |
| Sij-2 | Uzbekistan | CS9655 | 0.0844 | 0.0000 |
| St.yues-lik |  | V10537 | 0.0907 | 0.4545 |
| Kno-1 | USA | CS22401 | 0.0921 | 0.2000 |
| Ws-0 | Russia | N1595 | 0.0978 | 1.0000 |
| Asc-1 | UK | CS22163 | 0.0995 | 0.9500 |
| Amel-3 | Netherlands | V10530 | 0.1026 | 0.2727 |
| Sij-4 | Uzbekistan | CS9656 | 0.1040 | 0.6667 |
| Kil-0 | UK | CS6754 | 0.1065 | 0.1429 |
| Lac-4 | France | W40289 | 0.1060 | 0.0000 |
| **Col** | **USA** | **A22625** | **0.1093** | **0.5610** |
| Fet-6 | France | W40286 | 0.1100 | 0.3000 |
| No-0 | Germany | CS1394 | 0.1130 | 0.1000 |
| Pyl | France | V10370 | 0.1143 | 0.4000 |
| Tanz-1 | Tanzania | CS75924 | 0.1144 | 1.0000 |
| Mar2-4 | France | W40278 | 0.1166 | 0.9000 |
| Rsch-0 | Russia | CS6848 | 0.1173 | 0.6667 |
| Mog-12 | France | V40294 | 0.1228 | 1.0000 |
| Churchill |  | CS1072 | 0.1238 | 0.7000 |
| Sah-0 | Spain | CS6917 | 0.1242 | 0.6667 |
| O-17 |  | V10451 | 0.1247 | 0.1250 |
| Cal-0 | UK | CS6659 | 0.1285 | 0.0000 |
| Oerd-4 | Netherlands | W10040 | 0.1288 | 0.9000 |
| Marb-1 | Netherlands | CS75968 | 0.1293 | 0.8700 |
| Ovliel-1 | Netherlands | W10440 | 0.1298 | 0.8200 |
| Vliel-1 | Netherlands | V10438 | 0.1302 | 0.9000 |
| Oerd-2 | Netherlands | W10299 | 0.1315 | 0.8900 |
| Mog-1 | France | V40292 | 0.1317 | 0.6250 |
| Can-0 | Spain | CS2850 | 0.1321 | 0.3000 |
| Lm-2 | France | CS6784 | 0.1322 | 0.0000 |
| Polp-1 |  | V10536 | 0.1328 | 0.2500 |
| Ren-1 | France | CS22253 | 0.1331 | 0.7143 |
| Kz-2 | Kazaksthan | CS22436 | 0.1345 | 0.6250 |
| Boxmeer |  | W10545 | 0.1346 | 0.5000 |
| Csh-1 | USA | CS22419 | 0.1371 | 0.7143 |
| Hs-1 | USA | CS22351 | 0.1381 | 0.8000 |
| Rhen-4 | Netherlands | V10499 | 0.1393 | 0.9000 |
| Wei-1 | Switzerland | CS6182 | 0.1395 | 0.3333 |
| Ta-0 | Czech Rep | N1549 | 0.1429 | 0.5556 |
| Rhen-3 | Netherlands | V10498 | 0.1448 | 0.9500 |
| Ts-1 | Spain | A22647 | 0.1449 | 0.2500 |
| Borde | Germany | W10290 | 0.1493 | 0.0000 |
| Wag-11 | Netherlands | W10295 | 0.1508 | 0.8000 |
| Mir-0 | Italy | N1379 | 0.1520 | 0.3333 |
| Deil-1 | Netherlands | W10293 | 0.1520 | 0.8000 |
| Benk-3 | Netherlands | W10286 | 0.1521 | 0.0000 |
| Wt-4 | Germany | CS6895 | 0.1533 | 0.8000 |
| Wag-13 | Netherlands | V10428 | 0.1546 | 0.8182 |
| Fet-1 | France | W40284 | 0.1616 | 0.4000 |
| Lim | USA | CS8070 | 0.1525 | 0.3500 |
| Cam-3 | France | W40296 | 0.1526 | 0.3400 |
| Treb-1 |  | V10542 | 0.1605 | 0.3750 |
| **Mog-11** | **France** | **V40293** | **0.1664** | **0.1190** |
| Penz-2 |  | V10543 | 0.1665 | 0.9800 |
| Wt-5 | Germany | CS6896 | 0.1742 | 0.9580 |
| **Looe-2** | **UK** | **V10540** | **0.1756** | **0.1667** |
| **Bs-1** | **Switzerland** | **CS6627** | **0.1757** | **0.1111** |
| Lth-1 | USA | CS22363 | 0.1764 | 0.8000 |
| Be-0 | Germany | CS6613 | 0.1767 | 0.6000 |
| Driel-1 | Netherlands | CS75949 | 0.1965 | 0.7500 |
| **Nd-1** | **Germany** | **CS1636** | **0.2131** | **0.0791** |
| **Got-1** | **Germany** | **CS22277** | **0.2193** | **0.2378** |
| Blh-1 | Czech Rep | CS6645 | 0.2213 | 0.9700 |
| Tsu-1 | Japan | N1640 | 0.2221 | 0.5556 |
| Te-0 | Finland | CS6918 | 0.2299 | 0.7778 |
| Oy-0 | Norway | N1437 | 0.2316 | 0.7500 |
| **Sav-0** | **Czech Rep** | **N1514** | **0.2387** | **0.3333** |
| Kyo-1 | Japan | V10372 | 0.2514 | 0.7500 |
| **Wil-1** | **Russia** | **N1595** | **0.2659** | **0.0816** |
| Mib-3 | France | V40305 | 0.2916 | 1.0000 |

Name, Stock number (N, NASC stock center (<http://arabidopsis.info/>); A, ABRC stock center (http://abrc.osu.edu/); V, INRA Versailles collection (<http://dbsgap.versailles.inra.fr/vnat/)>; W, Wageningen university collection) and country of origin of the 82 accessions are reported. The 7 selected tolerant accessions and the reference accession Col-0 are displayed in blod. In the last 2 columns, the percentage of relative growth of the primary root in response to 150 mM NaCl (see Materials and Methods) and cotyledon albino rate under 200 mM NaCl are given.
