# Supplementary material for: Potassium Retention under Salt Stress Is Associated with Natural Variation in Salinity Tolerance among Arabidopsis Accessions
Source: PLoS One. 2015 May 19;10(5):e0124032. doi: 10.1371/journal.pone.0124032 (PMC4438003; doi:10.1371/journal.pone.0124032)
Supplement: S2 Table — (DOC) [file pone.0124032.s010.doc]

**S2**_**Table.doc Primers used for genome sequence analysis.**

| Gene name | Genbank accession number | Forward or reverse primer | Sequence (5’-3’) | Product  (bp) |
| --- | --- | --- | --- | --- |
| *AtSOS1* | AT2G01980 | Forward | AAACATTGAAGAATTGGTCGGC | 1490 bp |
|  |  | Reverse | GCCAGACCAATGCCTACACTAAAT |  |
|  |  | Forward 1 | AATTCCAGGACGGCGATTG | 1501 bp |
|  |  | Reverse 1 | CCAACAACAACAACACGCGA |  |
|  |  | Forward 2 | TCACGACCCAAATTTCTTACAGG | 1363 bp |
|  |  | Reverse 2 | TGTCGTGCAATTGTATGTGCG |  |
|  |  | Forward 3 | TTCTTCATTCTAAAGTTGTCCCACG | 1461 bp |
|  |  | Reverse 3 | AACCAATGCTGTTGCAGTCG |  |
|  |  | Forward 3-1 | CGTTATCGGTTGCTCTTCCTC | 744 bp |
|  |  | Reverse 3-1 | GATAATGCGGCGGGAGAT |  |
|  |  | Forward 4 | TGCGACTTGATTACAGATTCTATGG | 1271 bp |
|  |  | Reverse 4 | CTCAATTCTCATAGATCGTTCCTGA |  |
|  |  | Forward 4-1 | GCAACTCAGCATTTTCGGC | 680 bp |
|  |  | Reverse 4-1 | CATTACGAGTGATAGATAGGGAAAAG |  |
| *AtSOS2* | AT5G35410 | Forward | GACTACGCCCAATCGCAAT | 1331 bp |
|  |  | Reverse | AAAAGAATAACCCCGCAAGAC |  |
|  |  | Forward 1 | AGGTTTCGGATTTCGGACTC | 1457 bp |
|  |  | Reverse 1 | TAAGGCAGTCAAAACGGGC |  |
|  |  | Forward 2 | GCACTATTTGACAGGCGACAG | 1171 bp |
|  |  | Reverse 2 | CGCAGGACAAGTTTTGAGAAG |  |
| *AtSOS3* | AT5G24270 | Forward | TTTGCGTTTGGTTGTAGCGTG | 1647 bp |
|  |  | Reverse | GTATGTGAGATGGAGAGTCGCTATG |  |
| *AtNHX1* | AT5G27150 | Forward | AGGAATGGTTTCAGTGGACAGC | 1042 bp |
|  |  | Reverse | CCAATCGTAAAGCGAAGTAACC |  |
|  |  | Forward 1 | AAGAAGCAGTTTTTCCGCAAT | 1431 bp |
|  |  | Reverse 1 | ACGATAACGGAAAGACGAACG |  |
|  |  | Forward 2 | GTTGGAATGGATGCCTTGG | 655 bp |
|  |  | Reverse 2 | GGCGTTCTGGTGCGGTAAT |  |
|  |  | Forward 3-1 | CTTGTTTTCAGGTGTTTGGTATGC | 324 bp |
|  |  | Reverse 3-1 | CAGGAGGGTTTCTCTCAGTTGG |  |
|  |  | Forward 3-2 | CTTGTTTTCAGGTGTTTGGTATG | 517 bp |
|  |  | Reverse 3-2 | ATTACATACATCAATCAAACACTTCAA |  |
| *AtHKT1;1* | AT4G10310 | Forward | GAAAAAACAGGAATCGCTATCAT | 1091 bp |
|  |  | Reverse | CTCAGAGGTCCATTCAAAGGC |  |
|  |  | Forward 1 | CTCTCATCTACTCTCGGTTCGTC | 1530 bp |
|  |  | Reverse 1 | GGAGGAAGATACCTTGGAAACG |  |
|  |  | Forward 2 | CTTCAATGTAGTGGAGACTGGAG | 303 bp |
|  |  | Reverse 2 | TGTAGATTTTGCCTTTCGGTG |  |
|  |  | Forward 2-1 | CCCTTCAATGTAGTGGAGACTGGAG | 1284 bp |
|  |  | Reverse 2-2 | ATCGCTGATGTCCACACGCC |  |
|  |  | Forward 3-1 | ATTTGACAACCCAAGTATTCGTAA | 987 bp |
|  |  | Reverse 3-1 | ATCACTCACAGAAAAGGAATGCT |  |
