# Supplementary material for: Potassium Retention under Salt Stress Is Associated with Natural Variation in Salinity Tolerance among Arabidopsis Accessions
Source: PLoS One. 2015 May 19;10(5):e0124032. doi: 10.1371/journal.pone.0124032 (PMC4438003; doi:10.1371/journal.pone.0124032)
Supplement: S3 Table — (DOC) [file pone.0124032.s011.doc]

| Gene name | Genbank accession number | Forward/reverse primer | Sequence (5’-3’) | Product  (bp) |
| --- | --- | --- | --- | --- |
| *AtSOS1* | AT2G01980 | Forward | ATGATGGGACGGCGATTGT | 135 bp |
|  |  | Reverse | TGCCAAACGCCAGACCAAT |  |
| *AtSOS2* | AT5G35410 | Forward | TTGGTCTTGCGGGGTTATTC | 177 bp |
|  |  | Reverse | CGTGTTTTGGGATTGGGGT |  |
| *AtSOS3* | AT5G24270 | Forward | CGGAGGAATCTCTTCGCTG | 109 bp |
|  |  | Reverse | CGCTTGGATGGAAGACACC |  |
| *AtNHX1* | AT5G27150 | Forward | CTACCTATTACCGCACCAGAACG | 103 bp |
|  |  | Reverse | CTCAATGAACGAGTCTTGGTCC |  |
| *AtHKT1;1* | AT4G10310 | Forward | ACGGCGAGAGATGTTCTTAGTTCCAA | 111 bp |
|  |  | Reverse | CATGTTCTCATTCGTGGGGACAAAT |  |
| *AtHAK5* | AT4G13420 | Forward | CATCGCTGTTACCCTTGCCTTTAG | 172 bp |
|  |  | Reverse | TATGGAGCCAAAGACGACCAAGAA |  |
| *AtCHX17* | AT4G23700 | Forward | ATAGAAGCATCCCGAGGCATA | 225 bp |
|  |  | Reverse | ACGCACTGAGACACGGCTG |  |
| *AtKUP1* | AT2G30070 | Forward | GGCGATACCCGAGCCTGTGTT | 221 bp |
|  |  | Reverse | CCGCCAGGCAAAGACACATCA |  |
| *AtProDH1* | AT3G30775 | Forward | TGGTTTCGGTGTCGTTCTCG | 103 bp |
|  |  | Reverse | CTCTATCTTCCCGTTCTGTTTATCG |  |
| *AtP5CDH* | AT5G62530 | Forward | TGGTTTACCCGCTGAAGATGTGG | 96 bp |
|  |  | Reverse | GTGAAGAGGGTCATCCGTGGGT |  |
| *P5CR* | AT5G14800 | Forward | CTTTAGCCGATGGAGGAGTAGC | 127 bp |
|  |  | Reverse | TCTTTCAACACACCTGGATGCTT |  |
| *P5CS1* | AT2G39800 | Forward | GATTGGCTCTTGGTCGTTTAGG | 189 bp |
|  |  | Reverse | AAGCCTTCCCATCAAGTTCAGTC |  |
| *P5CS2* | AT3G55610 | Forward | CCTCCGCCAAGTGGACAGTG | 85 bp |
|  |  | Reverse | CTCAGCACCAAGTCCGAACCTAA |  |
| *AtCSD1* | AT1G08830 | Forward | GAAAACAAGTAACCAAAGAGAGACG | 161 bp |
|  |  | Reverse | CCCTCACTGCTGTTCAAAACTGC |  |
| *AtCAT2* | AT4G35090 | Forward | CTATCCGACCCACGCATCAC | 101 bp |
|  |  | Reverse | TTCAGACGGCTTGCCAGC |  |
| *AtAPX2* | AT3G09640 | Forward | TGGTCGGATGGGACTCAAT | 196 bp |
|  |  | Reverse | AAGAGCCTTGTCGGTTGGT |  |
| *AtZAT10* | AT1G27730 | Forward | AGGCTCTTACATCACCAAGATTAG | 237 bp |
|  |  | Reverse | TACACTTGTAGCTCAACTTCTCCA |  |
| *AtZAT12* | AT5G59820 | Forward | TGTCCCATATGTGGAGTGGA | 201 bp |
|  |  | Reverse | ATTGTCCACCATCCCTAGACT |  |
| *AtGORK* | AT5G37500 | Forward | TGTTCTTCAGTTCTTTGTTGCCTAT | 207 bp |
|  |  | Reverse | ACTTTGCGAACCCGAAATAGC |  |
| *AtACTIN2* | AT3G18780 | Forward | TCTTGTTCCAGCCCTCGTTTGTG | 170 bp |
|  |  | Reverse | TTCTTTGCTCATACGGTCAGCGATAC |  |

**S3**_**Table.doc Primers used for real-time PCR analyses.**
