# Supplementary material for: Potassium Retention under Salt Stress Is Associated with Natural Variation in Salinity Tolerance among Arabidopsis Accessions
Source: PLoS One. 2015 May 19;10(5):e0124032. doi: 10.1371/journal.pone.0124032 (PMC4438003; doi:10.1371/journal.pone.0124032)
Supplement: S4 Table — The expression levels of these genes normalized to ACTIN2 gene respectively were analyzed in Col-0 and the selected tolerant accessions exposed to 100 mM NaCl for 0, 3 and 6 h. Values given as mean ± SE (n = 3). (DOC) [file pone.0124032.s012.doc]

**S4**_**Table.doc Expression profile of *AtHAK5*, *AtCHX17*, *AtKUP1* and *AtGORK* gene.** The expression levels of these genes normalized to *ACTIN2* gene respectively were analyzed in Col-0 and the selected tolerant accessions exposed to 100 mM NaCl for 0, 3 and 6 h. Values given as mean ± SE (*n* = 3).

| Gene | Treatment (100 mM NaCl) | Col-0 | Bs-1 | Mog-11 | Looe-2 | Got-1 | Wil-1 | Nd-1 | Sav-0 |
| --- | --- | --- | --- | --- | --- | --- | --- | --- | --- |
| *AtHAK5* | 0 h | 0.05±0.01 | 0.69±0.06 | 0.38±0.02 | 1.18±0.03 | 0.77±0.05 | 0.03±0.00 | 0.18±0.01 | 0.39±0.03 |
| *AtHAK5* | 3 h | 0.27±0.03 | 1.44±0.24 | 4.55±0.43 | 2.54±0.12 | 2.46±1.40 | 1.23±0.04 | 0.67±0.21 | 0.08±0.00 |
| *AtHAK5* | 6 h | 0.06±0.01 | 0.68±0.06 | 0.74±0.06 | 0.12±0.04 | 1.39±0.14 | 0.38±0.12 | 0.29±0.02 | 0.24±0.02 |
| *AtCHX17* | 0 h | 0.63±0.07 | 0.63±0.02 | 1.26±0.12 | 1.49±0.13 | 0.50±0.02 | 1.38±0.03 | 1.13±0.06 | 0.34±0.04 |
| *AtCHX17* | 3 h | 0.32±0.02 | 0.18±0.01 | 0.65±0.04 | 0.62±0.04 | 0.26±0.03 | 0.25±0.10 | 0.85±0.07 | 0.06±0.01 |
| *AtCHX17* | 6 h | 0.40±0.06 | 0.64±0.06 | 1.61±0.11 | 1.24±0.08 | 1.29±0.04 | 1.63±0.09 | 0.82±0.09 | 0.38±0.03 |
| *AtKUP1* | 0 h | 0.78±0.07 | 0.63±0.03 | 0.71±0.05 | 0.83±0.08 | 0.58±0.05 | 0.56±0.02 | 0.73±0.03 | 0.71±0.07 |
| *AtKUP1* | 3 h | 0.45±0.04 | 0.55±0.03 | 1.76±0.04 | 1.68±0.06 | 0.46±0.08 | 2.77±0.02 | 0.41±0.04 | 1.74±0.02 |
| *AtKUP1* | 6 h | 1.48±0.13 | 2.03±0.23 | 1.40±0.11 | 1.36±0.12 | 1.12±0.15 | 0.65±0.05 | 1.34±0.16 | 1.81±0.20 |
| *AtGORK* | 0 h | 0.51±0.08 | 0.62±0.05 | 1.05±0.09 | 0.87±0.09 | 0.81±0.10 | 1.39±0.04 | 0.45±0.01 | 0.95±0.09 |
| *AtGORK* | 3 h | 0.65±0.08 | 0.64±0.07 | 0.81±0.10 | 0.50±0.07 | 0.37±0.04 | 0.65±0.10 | 0.69±0.02 | 0.75±0.09 |
| *AtGORK* | 6 h | 0.36±0.04 | 0.32±0.06 | 0.52±0.09 | 0.37±0.04 | 0.17±0.03 | 0.42±0.07 | 0.35±0.04 | 0.62±0.08 |
