# Supplementary material for: Potassium Retention under Salt Stress Is Associated with Natural Variation in Salinity Tolerance among Arabidopsis Accessions
Source: PLoS One. 2015 May 19;10(5):e0124032. doi: 10.1371/journal.pone.0124032 (PMC4438003; doi:10.1371/journal.pone.0124032)
Supplement: S5 Table — The expression levels of these genes normalized to ACTIN2 gene respectively were analyzed in Col-0 and the selected tolerant accessions exposed to 100 mM NaCl for 0, 3 and 6 h. Values given as mean ± SE (n = 3). (DOC) [file pone.0124032.s013.doc]

**S5**_**Table.doc Expression profile of *AtSOS1*, *AtSOS2*, *AtSOS3*, *AtNHX1* and *AtHKT1;1* gene.** The expression levels of these genes normalized to *ACTIN2* gene respectively were analyzed in Col-0 and the selected tolerant accessions exposed to 100 mM NaCl for 0, 3 and 6 h. Values given as mean ± SE (*n* = 3).

| Gene | Treatment (100 mM NaCl) | Col-0 | Bs-1 | Mog-11 | Looe-2 | Got-1 | Wil-1 | Nd-1 | Sav-0 |
| --- | --- | --- | --- | --- | --- | --- | --- | --- | --- |
| *AtSOS1* | 0 h | 1.16±0.04 | 0.76±0.12 | 0.80±0.17 | 1.39±0.30 | 0.78±0.14 | 0.74±0.04 | 0.51±0.07 | 0.51±0.06 |
| *AtSOS1* | 3 h | 1.57±0.27 | 1.40±0.15 | 0.92±0.13 | 1.68±0.28 | 1.03±0.14 | 1.17±0.17 | 0.93±0.08 | 1.10±0.16 |
| *AtSOS1* | 6 h | 1.40±0.28 | 1.62±0.21 | 0.85±0.10 | 1.26±0.17 | 1.37±0.20 | 1.29±0.14 | 1.06±0.20 | 1.33±0.21 |
| *AtSOS2* | 0 h | 0.41±0.30 | 0.62±0.02 | 0.63±0.20 | 1.08±0.23 | 0.36±0.10 | 0.30±0.07 | 0.44±0.05 | 0.41±0.07 |
| *AtSOS2* | 3 h | 0.64±0.12 | 0.35±0.09 | 0.48±0.08 | 0.46±0.07 | 0.29±0.02 | 0.40±0.07 | 0.24±0.05 | 0.43±0.05 |
| *AtSOS2* | 6 h | 0.35±0.07 | 0.51±0.07 | 0.40±0.07 | 0.33±0.05 | 0.33±0.06 | 0.44±0.04 | 0.38±0.06 | 0.59±0.07 |
| *AtSOS3* | 0 h | 3.76±0.51 | 2.38±0.13 | 3.18±0.21 | 2.10±0.09 | 2.37±0.15 | 2.73±0.07 | 1.69±0.06 | 1.22±0.04 |
| *AtSOS3* | 3 h | 3.55±0.55 | 3.03±0.39 | 5.07±0.68 | 2.48±0.34 | 2.35±0.14 | 2.79±0.40 | 2.50±0.31 | 2.47±0.23 |
| *AtSOS3* | 6 h | 4.69±0.94 | 5.14±0.63 | 3.06±0.54 | 4.60±0.23 | 4.67±0.65 | 4.62±0.44 | 3.61±0.57 | 5.41±0.52 |
| *AtNHX1* | 0 h | 1.11±0.14 | 0.68±0.03 | 0.53±0.08 | 0.80±0.07 | 0.69±0.04 | 0.96±0.09 | 0.67±0.03 | 0.71±0.06 |
| *AtNHX1* | 3 h | 2.98±0.07 | 2.80±0.13 | 2.21±0.12 | 2.71±0.15 | 3.20±0.16 | 2.73±0.26 | 2.81±0.20 | 2.20±0.17 |
| *AtNHX1* | 6 h | 1.29±0.27 | 1.60±0.11 | 1.52±0.09 | 1.79±0.06 | 1.92±0.13 | 1.70±0.15 | 1.64±0.11 | 1.72±0.04 |
| *AtHKT1;1* | 0 h | 0.46±0.02 | 0.62±0.07 | 0.06±0.00 | 0.24±0.02 | 0.42±0.03 | 0.49±0.04 | 1.00±0.06 | 0.16±0.02 |
| *AtHKT1;1* | 3 h | 3.09±0.48 | 0.56±0.07 | 0.86±0.12 | 0.35±0.07 | 1.09±0.06 | 2.58±0.36 | 1.06±0.12 | 0.76±0.09 |
| *AtHKT1;1* | 6 h | 2.31±0.45 | 0.42±0.06 | 0.50±0.06 | 0.20±0.02 | 1.02±0.12 | 0.91±0.09 | 1.03±0.15 | 0.78±0.11 |
