# Supplementary material for: Potassium Retention under Salt Stress Is Associated with Natural Variation in Salinity Tolerance among Arabidopsis Accessions
Source: PLoS One. 2015 May 19;10(5):e0124032. doi: 10.1371/journal.pone.0124032 (PMC4438003; doi:10.1371/journal.pone.0124032)
Supplement: S6 Table — (DOC) [file pone.0124032.s014.doc]

**S6**_**Table.doc Linear correlation (*r^2^*) between NaCl-induced net K^+^ flux (100 mM NaCl) and the content of compatible solutes determined under 200 mM NaCl stress in this study.**

| Parameter | NaCl-induced net K^+^ flux^a^ | | | | | | | |
| --- | --- | --- | --- | --- | --- | --- | --- | --- |
| Accession | Col-0 | Bs-1 | Mog-11 | Looe-2 | Got-1 | Wil-1 | Nd-1 | Sav-0 |
| Proline content | 0.99* | 0.98* | 0.99* | 0.98* | 0.37 | 0.99* | 0.99* | 0.26 |
| Soluble protein content | 0.84 | 0.80 | 0.74 | 0.87 | 0.76 | 0.88 | 0.45 | 0.78 |
| Soluble sugar content | 0.78 | 0.69 | 0.92 | 0.99* | 0.97* | 0.73 | 0.97* | 0.99* |

^a^ Significant at * P <0.05.
